# Large-scale in-silico analysis of CSF dynamics within the subarachnoid space of the optic nerve

**DOI:** 10.1186/s12987-024-00518-8

**Published:** 2024-02-28

**Authors:** Diego Rossinelli, Gilles Fourestey, Hanspeter Esriel Killer, Albert Neutzner, Gianluca Iaccarino, Luca Remonda, Jatta Berberat

**Affiliations:** 1 Institute for Computational and Mathematical Engineering (ICME), Center for Turbulence Research, Stanford University, Stanford, CA 94305-3024 USA; 2 Institute of Neuroradiology, Kantonsspital Aarau, 5000 Aarau, Switzerland; 3 Scientific IT & Application Support (SCITAS), Swiss Federal Institute of Technology Lausanne (EPFL), Lausanne, Switzerland; 4 Department of Biomedicine, University of Basel, Basel, Switzerland; 5 Geriatric Psychiatry, Department of Psychiatry, University Hospitals of Geneva, University of Geneva, Geneva, Switzerland

**Keywords:** Cerebrospinal fluid, Subarachnoid space, Optic nerve, Intracranial pressure, Computational fluid dynamics, Optic nerve compartment syndrome, Idiopathic intracranial hypertension, Homeostasis, Dispersion, Normal-Tension Glaucoma

## Abstract

**Background:**

Impaired cerebrospinal fluid (CSF) dynamics is involved in the pathophysiology of neurodegenerative diseases of the central nervous system and the optic nerve (ON), including Alzheimer’s and Parkinson’s disease, as well as frontotemporal dementia. The smallness and intricate architecture of the optic nerve subarachnoid space (ONSAS) hamper accurate measurements of CSF dynamics in this space, and effects of geometrical changes due to pathophysiological processes remain unclear. The aim of this study is to investigate CSF dynamics and its response to structural alterations of the ONSAS, from first principles, with supercomputers.

**Methods:**

Large-scale *in-silico* investigations were performed by means of computational fluid dynamics (CFD) analysis. High-order direct numerical simulations (DNS) have been carried out on ONSAS geometry at a resolution of 1.625 μm/pixel. Morphological changes on the ONSAS microstructure have been examined in relation to CSF pressure gradient (CSFPG) and wall strain rate, a quantitative proxy for mass transfer of solutes.

**Results:**

A physiological flow speed of 0.5 mm/s is achieved by imposing a hydrostatic pressure gradient of 0.37–0.67 Pa/mm across the ONSAS structure. At constant volumetric rate, the relationship between pressure gradient and CSF-accessible volume is well captured by an exponential curve. The ONSAS microstructure exhibits superior mass transfer compared to other geometrical shapes considered. An ONSAS featuring no microstructure displays a threefold smaller surface area, and a 17-fold decrease in mass transfer rate. Moreover, ONSAS trabeculae seem key players in mass transfer.

**Conclusions:**

The present analysis suggests that a pressure drop of 0.1–0.2 mmHg over 4 cm is sufficient to steadily drive CSF through the entire subarachnoid space. Despite low hydraulic resistance, great heterogeneity in flow speeds puts certain areas of the ONSAS at risk of stagnation. Alterations of the ONSAS architecture aimed at mimicking pathological conditions highlight direct relationships between CSF volume and drainage capability. Compared to the morphological manipulations considered herein, the original ONSAS architecture seems optimized towards providing maximum mass transfer across a wide range of pressure gradients and volumetric rates, with emphasis on trabecular structures. This might shed light on pathophysiological processes leading to damage associated with insufficient CSF flow in patients with optic nerve compartment syndrome.

**Supplementary Information:**

The online version contains supplementary material available at 10.1186/s12987-024-00518-8.

## Introduction

The central nervous system is surrounded by the three-layered meninges. The outermost dura mater is a thick collagenous structure enclosing the central nervous system (CNS), thereby providing structural support and physical protection. The adjacent delicate arachnoid and pia mater enclose the subarachnoid space (SAS), forming an interface to the neuronal tissue underneath. The SAS is constantly perfused with CSF which is produced in the choroid plexus. From there, it distributes throughout the ventricles and around the neuronal tissue. Drainage of CSF through dural lymphatics and arachnoid granulations into the venous system balances its production and is thought to keep the pressure in the subarachnoid space of the optic nerve in check [1]. Besides providing a fluid cushion for the brain, CSF removes waste products such as amyloid beta, and supplies nutrients to the neuronal tissue [2]. These are critical activities as the latter is both metabolically very demanding and lacks energy storage mechanisms [30]. Furthermore, the absence of a classical lymphatic system in the CNS [41, 42] suggests that CSF plays an important role in maintaining homeostasis. Distribution of hormones and other signaling molecules is indeed mediated by CSF flow. In addition, within the neuronal parenchyma the so-called glymphatic system [3] is thought to be involved in the clearance of interstitial fluid. Moreover, the CSF flow allows the surveillance of the brain by immune cells resident in the dura and draining lymph nodes.

The optic nerve is an extension of the telencephalon and is, like the brain, surrounded by cerebrospinal fluid. The ONSAS constitutes the microenvironment for the optic nerve. This space is not empty but bridged by a complex network of trabeculae and septae, both of which are covered with layers of meningothelial cells.

Given such important roles of the CSF, it is not surprising that an impairment of its dynamics is linked to a range of diseases of the CNS [4], both in the brain and the optic nerve, such as idiopathic intracranial hypertension (IIH) [5], papilledema [6], hydrocephalus, normal tension glaucoma (NTG) [7], and possibly congenital glaucoma [38]. Detailed information about CSF dynamics in the ONSAS can contribute to a better understanding of optic nerve diseases like IIH, the translaminar pressure gradient in NTG, and spaceflight-associated neuro-ocular syndrome (SANS). Papilledema is characterized by progressive loss of visual field and visual acuity potentially leading to blindness due to axonal degeneration [8]. The current standard therapeutic treatment is the reduction of intracranial pressure either with medication or with surgical procedures such as shunts or optic nerve sheath fenestration. Despite successful ICP lowering, visual field loss has been reported [9]. Therefore, a deeper understanding of the detailed CSF dynamics within the ONSAS is important.

By experimentally observing hydrostatic continuity, the hypothesis that the intracranial pressure is directly transmitted in the ONSAS has been elaborated in several studies [10] and literature surveys [11, 12]. This hypothesis has been challenged by the fact that the CSF in the ONSAS can become compartmentalized [13, 14] and exhibit heterogeneous flow pattern [13, 14] thereby affecting CSFPG and in turn the retrolaminar tissue pressure. Compartmentalisation may increase flow resistance and develop a pressure drop across the ONSAS. If such a pressure drop is significant, ICP cannot be transmitted without losses to the retrolaminar subarachnoid space pressure. While measuring in-vivo CSF flow ratios within the SAS in the ON and the brain is attainable with imaging [15, 16], direct measurement of CSFP in living humans is not possible. The hypothesis that CSFP in the human optic nerve equals ICP largely remains speculative.

Several computational investigations of cerebrospinal fluid dynamics have been carried out in the past. Computational Fluid Dynamics (CFD) has been used to model CSF in the brain SAS relying on MRI velocimetry imaging [17, 18]. The geometrical details were not taken explicitly in consideration, rather they were sub-grid modeled as a porous anisotropic media. Other computational efforts aimed at better understanding the CSF dynamics in the spinal SAS [19]. A closely related question that has not yet been answered is how the detailed ONSAS geometry affects the CSF dynamics and hence CSFPG. Several studies modeled solute dispersion within the perivascular spaces (PVS) [44–47] mostly with idealized geometries, never fully based on 3D imaging, and not always with reconcilable conclusions.

A recent study on the morphometry of the ONSAS [20] estimated the area of the ONSAS structure interfacing the CSF. The measurement aimed at providing insight about the contribution of the meningeal layer to the biochemical CSF homeostasis.

The present work is oriented towards answering the following questions: (a) *Can large-scale in-silico investigations help clarify the relationship between CSFP and ICP? (b) Do small changes in the ONSAS structure lead to equally small changes in the CSF dynamics? (c) Are there parts of the ONSAS microstructure that contribute to mass transfer more than others, even if they expose comparable surface area?*

In turn, we report herein what might be the *first micrometer-resolution in-silico study of the CSF dynamics within whole-slice sections of the human ONSAS*. This is achieved by leveraging the best contemporary sensing and computing technology available at the present time. Specifically, high-volume high-quality signals were acquired ex-vivo from human specimen at a synchrotron imaging facility, thus bypassing any geometrical idealization and uncertainties related to in-vivo modalities and animal models, and computational workloads were carefully mapped to contemporary supercomputers by discretizing the incompressible formulation of the Navier–Stokes equations with very accurate numerical schemes. Such simulations capture the fluid dynamics and the effect of the morphology from first principles, as opposed to reduced-order modeling of sorts, and synthesized geometries. This is in strong contrast against most of the in-silico analysis reported insofar [30].

This approach enabled us to carry out a quantitative analysis of the CSFP response to ICP, as well as a quantitative analysis of how ONSAS geometrical features affect CSF dynamics and mass transfer.

## Methods

This section describes methodology used to carry out the simulations, alongside with the pre-processing stages required to manipulate the captured geometry.

### Sample Preparation and Imaging

Post-mortem, a single healthy human optic nerve of about 6 mm × 8 mm × 10 mm, was harvested from an 85-year-old male (EKNZ-2021–00031). Two whole-slice specimens were taken from the optic nerve: one in the bulbar section, and one in the intraorbital section. For both specimens, the thickness was approximately 0.75 mm. Osmium tetroxide was employed for sample fixation. Imaging was carried out with synchrotron radiation-based micro computed tomography (SRμCT) at the TOMCAT beamline of the Swiss Light Source at Paul Scherrer Institute (PSI) at a resolution of 0.375 μm/pixel and 3.75 μm/pixel. Further details can be found in [20].

### Image processing

To segment the SR*μ*CT images into CSF-filled subarachnoid space, as well as the ONSAS microstructure, image enhancement was necessary. Using high-order digital filters, the grayscale signal was denoised and CSF signal was transformed into a much narrower range of pixel intensity and resampled within an image stack of approximately 4000 × 4000 × 1000 pixels. Next, a smooth CSF space segment foreground was obtained through morphological operations (opening/closing) coupled with manipulations of connected components. We refer to [20] for further details.

### Computational fluid dynamics

#### Geometry

**Fig. 1:**
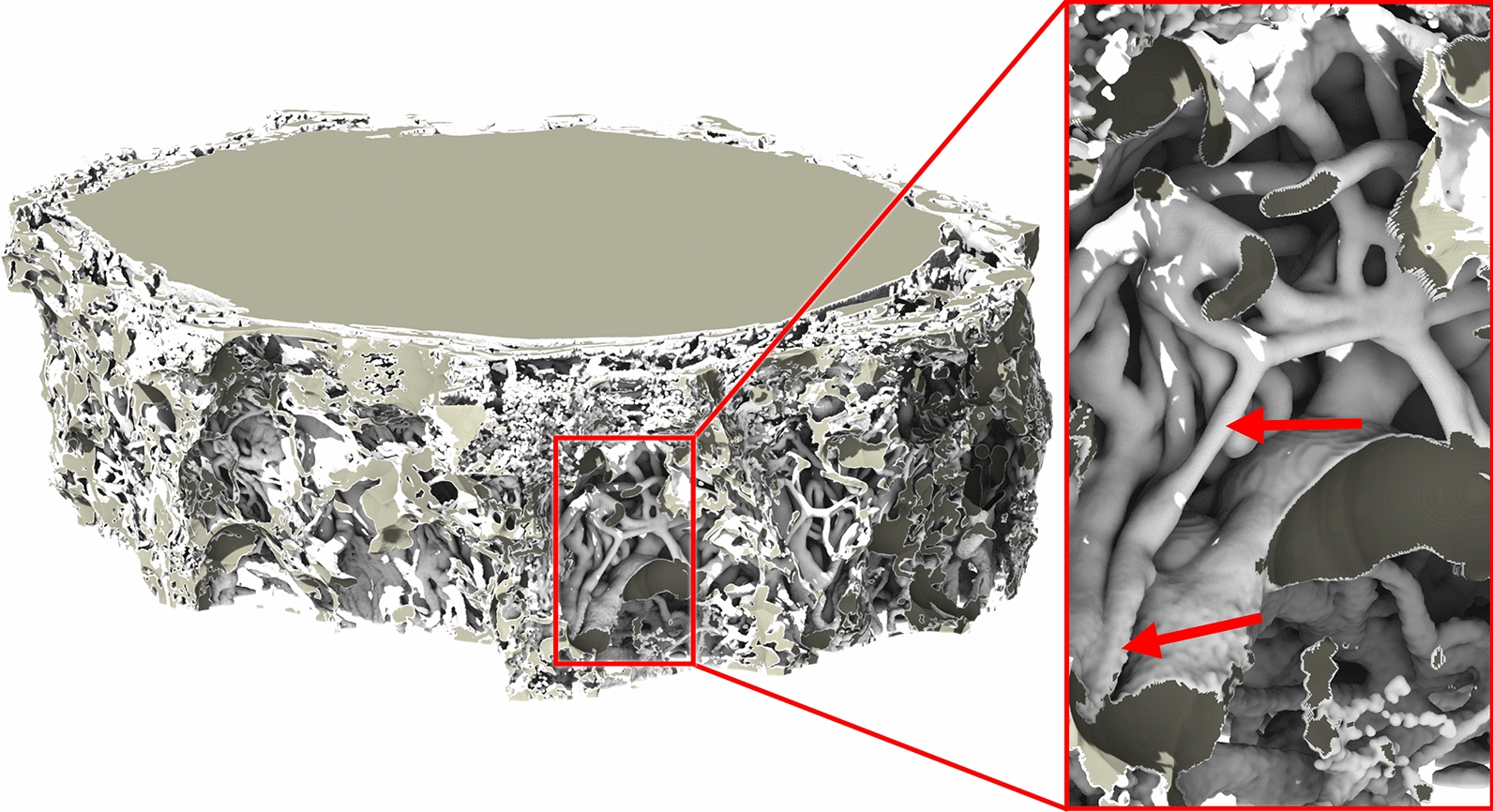
Seamless stitching of the ONSAS coarse and high-resolution segments of *Dataset1* (bulbar section). The coarse-to-fine transition is perceived by differences in smoothness (red arrows in the right picture)

**Fig. 2:**
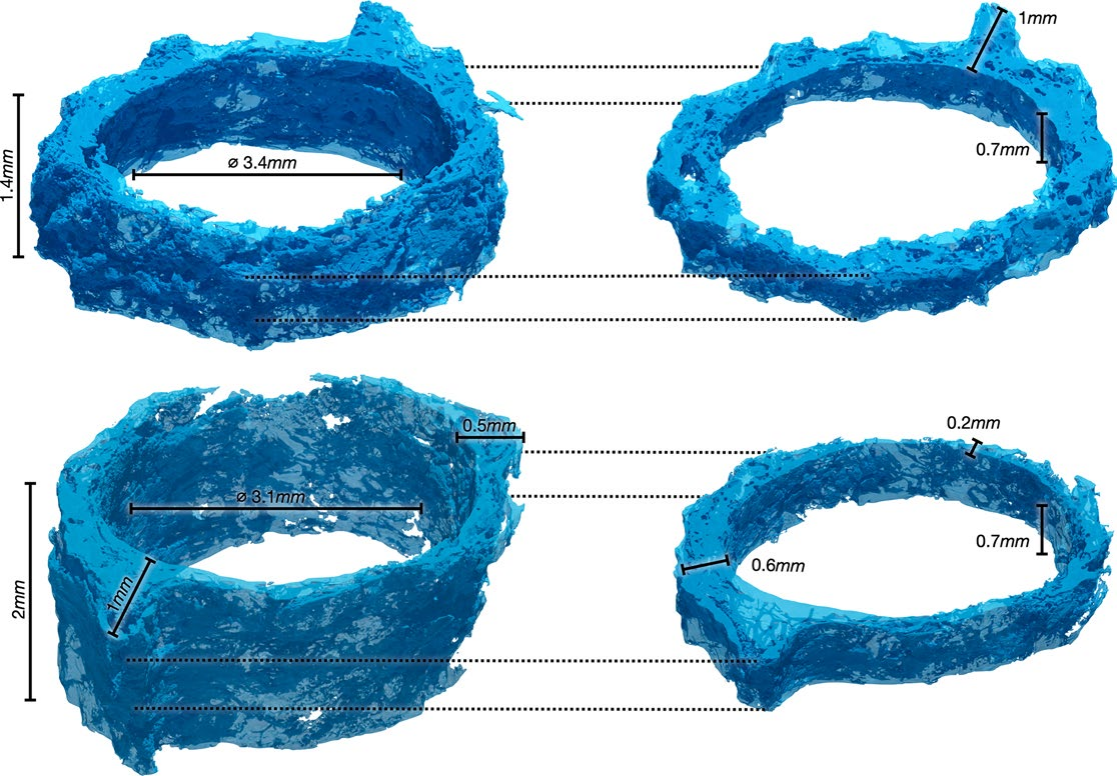
Fluid phase of the computational domains (translucent blue) for *Dataset1* (retrobulbar, top) and *Dataset2* (midorbital, bottom) of the CFD simulations (left), corresponding to the CSF space. The high-resolution part of the CSF image segment (right) is embedded at the middle of the computational domain

Following the same convention as in [20], we will refer to bulbar geometry as Dataset1 and the intraorbital geometry as Dataset2. In order to capture realistic inlet and outlet flow conditions, we extended both geometries by taking advantage of the larger field of view of the coarser SR*μ*CT tiles generated for previewing purposes, and acquired at a 10:1 resolution (3.25* μm/pix*). We segmented the coarse tiles, upsampled and merged them with the high-resolution segment foregrounds, thus obtaining a 1:2 foreground extension in the longitudinal direction. For the quantitative analysis of the CFD results we ensured to examine the CSF flow field only in the high-resolution image segment to rule out any spurious effects of the limited size of the domain. Figures 1 and 2 show how the high-resolution section is seamlessly embedded in the domain, both in terms of structure and fluid phases.

#### Reynolds number

Due to the multiscale features of the ONSAS architecture, assigning a single characteristic length L, as well as a single characteristic velocity U, to this flow problem is not obvious. For an average “macroscopic CSF space thickness” of 0.2 mm, the Reynolds number corresponds to Re_D_ ~ 0.1 (See ONSAS “thickness” in Fig. 2). Further intricacies of estimating the Reynolds number arise from the limited resolution of the MRI data regarding the CSF flow speed, and from the fact that we are examining two sections of the same optic nerve. If the average speed is assumed to be 0.5 mm/s at the intraorbital region, then due to mass conservation the average velocity in the bulbar section will be significantly lower, because of its larger cross-sectional area. The smallest Re is 0.03 because of the area ratio of about 1.8:1 between the bulbar and intraorbital cross-sections.

#### Computational modeling

CSF is modeled as a Newtonian fluid [21] with a density of 1 g/ml [22] and a viscosity of 1 mPa/s [23]. We assume that CSF streams through the ONSAS at 0.5 mm/s on average [15]. The ONSAS geometry is modeled as a stiff, non-deforming material for computational efficiency reasons. The flow problem is governed by the Navier–Stokes equation:

$$\frac{\partial u}{{\partial t}} + \left( {u \cdot \nabla } \right)u = - \frac{1}{\rho }\nabla p + v\Delta u$$

where u is the flow velocity and p is the total pressure of the flow. The flow is assumed to be incompressible:

$$\nabla \cdot u = 0$$

We assume that a hydrostatic pressure gradient drives the CSF through the ONSAS. Accordingly, we impose a hydrostatic pressure drop between the two ends of the optic nerve in our geometry.

$$p\left( X \right) = p1,\,{\rm X} \in \Gamma_{{{\text{Outlet}}}}$$

$$p\left( X \right) = p_{0} ,{\rm X} \in \Gamma_{{{\text{inlet}}}}$$

Such a model ignores the pulsating component driving the CSF flow. However, two remarks must be made. Firstly, a pulsatile flow below Re ~ 10, transiency without compliance is not expected to influence the spatial flow structure: if there is a clear separation within the scales, as expected, then the transient flow field can be accurately reconstructed by a tensor product between spatial and temporal basis functions. Secondly, there is experimental evidence of bulk flow by CSF through the SAS [44]. Thirdly, numerical evidence indicates that even if bulk flow is very small, it significantly contributes to mass transfer, whereas pure oscillatory flows minimally contribute or do not contribute at all [43, 48]. This may suggest that pulsatility does not play a central role to dispersion.

The boundary condition at the wall, i.e., the meningeal layer surface, is modeled as no-slip, no-through:

$$u\left( {\rm X} \right) = 0,{\rm X} \in \Gamma$$

We expect the no-slip condition to overestimate the pressure drop to attain a prescribed velocity, compared to fluid slip conditions. Conversely, the no-through condition is expected to underestimate the total pressure drop to attain the target flow rate, as the flow does not perfuse CSF through the structure.

To rule out any doubts about the relevance of the convection term and transiency for the specific flow problem at hand, we employ a fully transient CFD pseudo-spectral solver for incompressible flow and carry out the calculations until we reach a steady state: simulations complete when the temporal difference between the flow fields of two consecutive timesteps is below 10^–6^, in a relative L_∞_ sense:

$$\frac{{\left| {u_{i}^{t + 1} - u_{i}^{t} } \right|_{\infty } }}{{\max \left( {10^{ - 6} ,\left| {u_{i}^{t} } \right|_{\infty } } \right)}} < 10^{ - 6}$$

for all individual scalar entries of the flow field.

To solve the Navier–Stokes Equations we rely on a high-order pseudospectral scheme combined with an immersed boundary (IB) technique [24]. Structures are immersed in the fluid domain, resulting in a single grid encompassing both the flow and the fixated tissues. Such schemes lead to accurate results while providing a simple way of considering complex geometries [20, 26, 30]. In the context of computational investigation of CSF, IB approaches are regarded as useful to deal with complex geometries [30], such as herein. The reader is encouraged to read the detailed review on this technique outlined by Mittal and Iaccarino [25]. IB techniques are not exposed to the same accuracy concerns of CFD approaches relying on meshing, such as finite volume methods on unstructured meshes. Table 1 reports on the system sizes considered in the present work. Details about the employed solver, verification, validation [27–29], dimensionalisation, and spatial convergence are described in Additional file 3.

**Table 1:** System size of the high-resolution geometry, and the extended domains

		System size	CFD working footprint
Dataset1	ROI	3584 × 3968 × 448 cells	–
	Overall	3584 × 3968 × 864 cells	1.5 TB
Dataset2	ROI	3240 × 3240 × 452 cells	–
	Overall	3240 × 3240 × 1280 cells	1.6 TB

One could argue that high-order DNS is an overly sophisticated approach for the flow problems at hand, and a lower order discretization should be used instead. The reasoning behind this argument would be that the numerical dissipation of the associated schemes is tolerable as the flow does not exhibit sharp features. However, the numerical dissipation of low-order schemes is unphysical, their transfer function is qualitatively very different from the one of a diffusion process, as the former notches certain low frequencies and does not attenuate enough some other high frequencies, possibly leading to a substantial corruption of scales.

Regarding spatial convergence analysis on the large-scale ONSAS CFD, a resolution study starting with 3584 × 3968 × 864 computational elements (totaling 1.5 TB of aggregate RAM footprint) is not practical. However, within Additional file 3, we provide a grid convergence study on a coarser version of the image segment, increasing the grid resolution up to the one used for production simulations.

#### System sizes and computational resources

Table 1 shows the system size and the aggregate RAM footprint of the simulation domains. Each DNS is distributed across the computational nodes of *Helvetios.* A single DNS took about 144 h on 32 nodes (35 TFLOP/s, 2.9 TB/s, per DNS). Overall, more than 10,000 node hours have been spent on the present computational investigation.

### Quantitative Investigation

#### Difference between CSFP and ICP

Hydrostatic pressure typically refers to a difference in elevation, due to gravity. However, static pressure can be defined as any solution of a Laplace equation (i.e. a stationary scalar field featuring zero curvature everywhere). In the simulation we control the difference in static pressure between the inlet and outlet, to drive the flow. From the CFD results we measure the drop in total pressure $\Delta p = p0 - p1$ that matches the target average velocity, over a plane orthogonal to the direction of the optic nerve:

$$\int_{{\Gamma {\text{outlet}}}} {u \cdot dA = \dot{V}} = \int_{{\Gamma {\text{outlet}}}} {0.5\left[ {{{mm} \mathord{\left/ {\vphantom {{mm} s}} \right. \kern-0pt} s}} \right]} dA$$

where the region of the integration is the area of the section (referred as “cross-sectional” area). MRI-based measurements of CSF flow in healthy human subjects suggest an average flow speed between 0.163 mm/s up to 0.733 mm/s [15], conveying large interindividual variability. The target speed in this work is chosen to be 0.5 mm/s, and a Reynolds number of 0.1. We numerically observed that the non-linear terms in the NSE do not play an important role, as the inertial forces are small. In turn, linear superposition principles apply, allowing us to conveniently rescale the flow field to any desired velocity in the 0.1 mm/s–1 mm/s range.

### CSF homeostasis

We aim at estimating the contribution of the meningeal layer to CSF homeostasis, supply of nutrients, and removal of toxic compounds. In the present work, such phenomena are modeled in terms of mass transfer between a fluid flow (forced convection) and a bounding surface. Specifically, we assume that dispersion of solutes is obtained by combining passive advection with diffusion. This model is characterized by the Peclet number, a ratio between the timescales of the two processes. Since for the mass transfer problem in consideration, geometrical features are intrinsic to advection, the focus is on the latter. Here, the wall shear stress is related to the friction coefficient in the same way as mass transfer is related to the Sherwood number. Both quantities obey the same governing dimensionless equation and are said to be *analogous*. For more details about boundary layer analogies we refer to [31].

As we assume constant viscosity of the CSF, the calculation of wall shear stress (dynamic quantity) is proportional to the strain rate (kinematic quantity). Accordingly, for a qualitative comparison of different geometries we restrain our focus to the strain rate tensor at the meningeal surface, evaluated along its normal direction:

$${\text{t}} = \frac{1}{2}\left( {\nabla u + \nabla u^{\rm T} } \right) \cdot n$$

The magnitude of the tangential component of the strain rate then is integrated over the whole meningeal surface:

$$K = \int_{\Gamma } {\left| {t - \left\langle {t,n} \right\rangle n} \right|} d\Gamma$$

K represents a total mass transfer between structure and flow, mediated by passive transport. From its unit of measure, [m^2^/s], we note that K is a mass diffusivity that depends on the geometry of the ONSAS. We employ this quantity as a metric to compare how different ONSAS geometries may contribute to the CSF homeostasis. For a given geometry, this metric leads to lower values for slower flows. Moreover, lower values of K are also obtained for geometries leading to comparable flow speeds but smaller area. Although it cannot be considered a universal proxy for nutrient uptake, it enables the quantification and identification of stagnating regions that may impair drainage.

### Response to morphological changes

Morphological operations represent a viable tool to carry out a sensitivity analysis on global flow quantities against geometrical manipulations. Specifically, we employ morphological opening and closing operations to increase/decrease the ONSAS volume filled with CSF and conversely decrease/increase the trabecular thickness.

Previous work relied on morphological operations to demonstrate the homeostatic contribution of ONSAS microstructure in terms of purely geometrical features [20]. Similarly, being able to digitally remove the ONSAS trabecular microstructure allows us to quantify its contribution.

In terms of spatial fidelity of pathological morphogenesis, we are unaware of any research assessing such operations. Physiologically accurate modeling of cellular growth, however, is beyond the goal of the present work. We leverage the properties of morphological operations to obtain very different topologies while well preserving shape features, retaining comparable area, and keeping volume under control. A morphological closing allows to seal partially confining walls and create compartments without altering the wall thickness. At the same time, morphological operations allowed us to completely remove trabeculae to quantify their contribution, in a similar fashion as previously reported [20].

In a broader context, any geometrical manipulation of a surface can be decomposed in normal and tangential contributions. Morphological operations are in this regard key, as they represent changes in the tangential direction. Manipulations normal to the surface are more challenging to control as they may lead to drastic changes in curvature and directly impact volume.

**Fig. 3:**
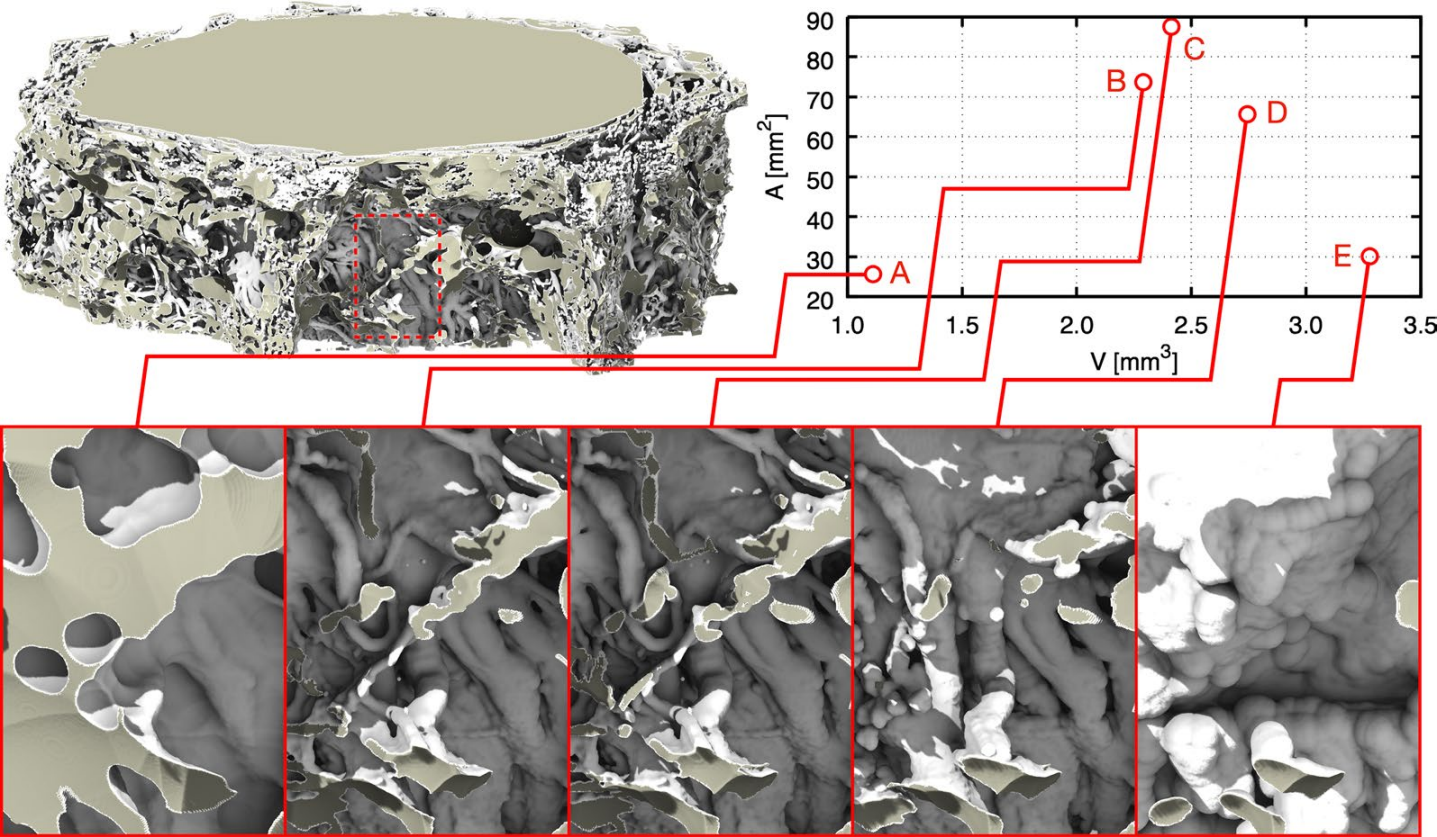
Original geometry (top left), alterations of the area and volume of the high-resolution section, caused by morphological manipulations (top right), as well as visual analysis of the morphological changes on the ONSAS microstructure (bottom) of a portion of the geometry (dashed rectangle, top left)

Overall, we consider 5 configurations of *Dataset1*:

- Morphological closing on the structure by 40 μm, to retain CSF-filled pockets (Fig. 3A)
- Morphological closing on the structure by 10 μm, to coalesce thin trabeculae (Fig. 3B)
- No morphological operations (Fig. 3C)
- Morphological opening on the structure by 10 μm, to break thin trabeculae (Fig. 3D), and
- Morphological opening on the structure by 50 μm, to remove the latter (Fig. 3E).

A closing operation consolidates the connection between trabeculae and coalesces the thinnest structures into fewer ones. An opening operation tends to separate trabeculae and break apart or completely remove the thinnest structures. While closing/opening will add/remove volume to the structure, respectively, it tends to decrease surface area, as observed in the plot of Fig. 3. For a detailed quantification of morphological operations on prototypical geometries, the reader is encouraged to consult the Additional file 1: Material (S1).

Figure 3 provides an overview of the manipulations. We note how the leftmost configuration severely challenges the CSF route, and the rightmost configuration features essentially no ONSAS microstructure. Table 2 summarizes the considered morphological changes in terms of area and volume gain/loss. The reader is encouraged to consult the Additional file 2 for a detailed visual assessment of each configuration.

**Table 2:** Changes in area and volume in response to the morphological operations, overall and for the high-resolution region of interest

Morphological operation on trabeculae	Volume		Area	
	Overall [mm^3^]	ROI [mm^3^]	Overall [mm^2^]	ROI [mm^2^]
Closing 40 μm	2.5	1.1	54.4	25.7
Closing 10 μm	5.0	2.3	150.2	73.7
Original	5.2	2.4	174.9	87.6
Opening 10 μm	5.8	2.7	132.8	65.6
Opening 50 μm	6.9	3.3	62.9	30.1

## Results

### CSF dynamics

**Fig. 4:**
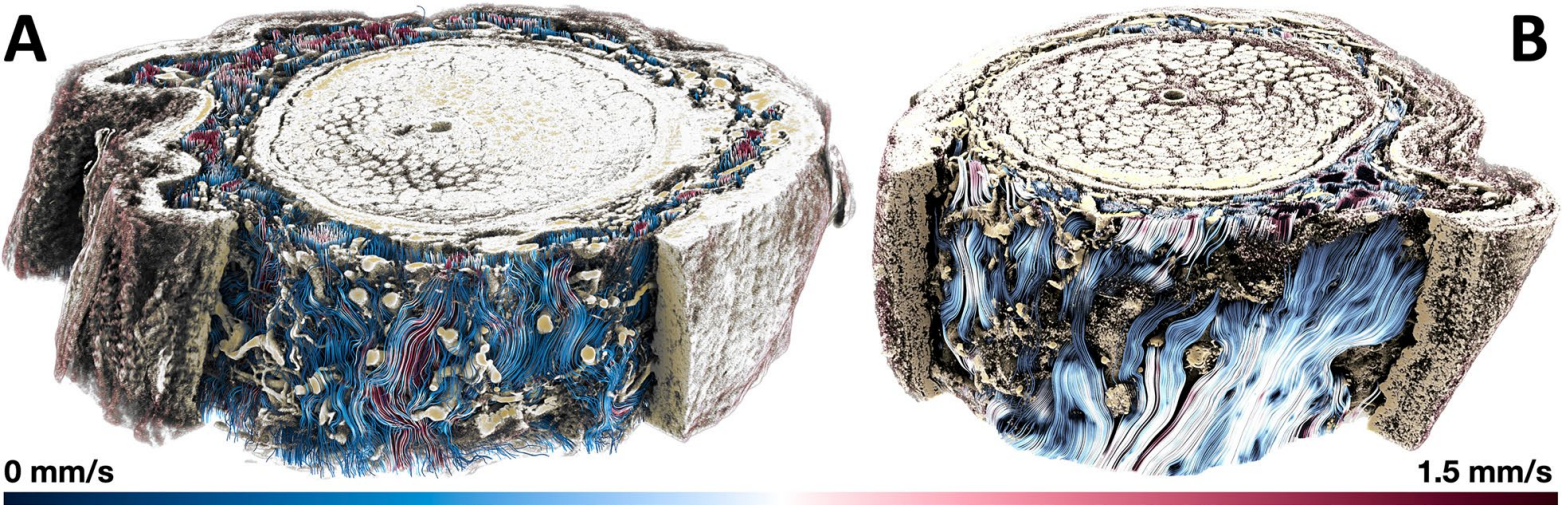
Streamlines illustrating CFD results in relation to the SR*μ*CT stitched grayscale signal of the extended *Dataset1* (A, bulbar section) and *Dataset2* (B, intraorbital). About a third of the dura was computationally removed to reveal streamlines

We rely on flow streamlines to understand how CSF moves through the ONSAS. In Fig. 4 we illustrate CSF dynamics across the two ON segments. We find that flow speeds are variable and range from zero to 1.5 mm/s. The thicker ONSAS of *Dataset1* gives space to the CSF to find multiple routes. In contrast, the ONSAS in *Dataset2* is substantially more confined and with fewer trabeculae. We also observe that CSF is more exposed to stagnation in *Dataset2*, in the region where the ONSAS is almost collapsing. More details can be found in the Additional file 7.

**Fig. 5:**
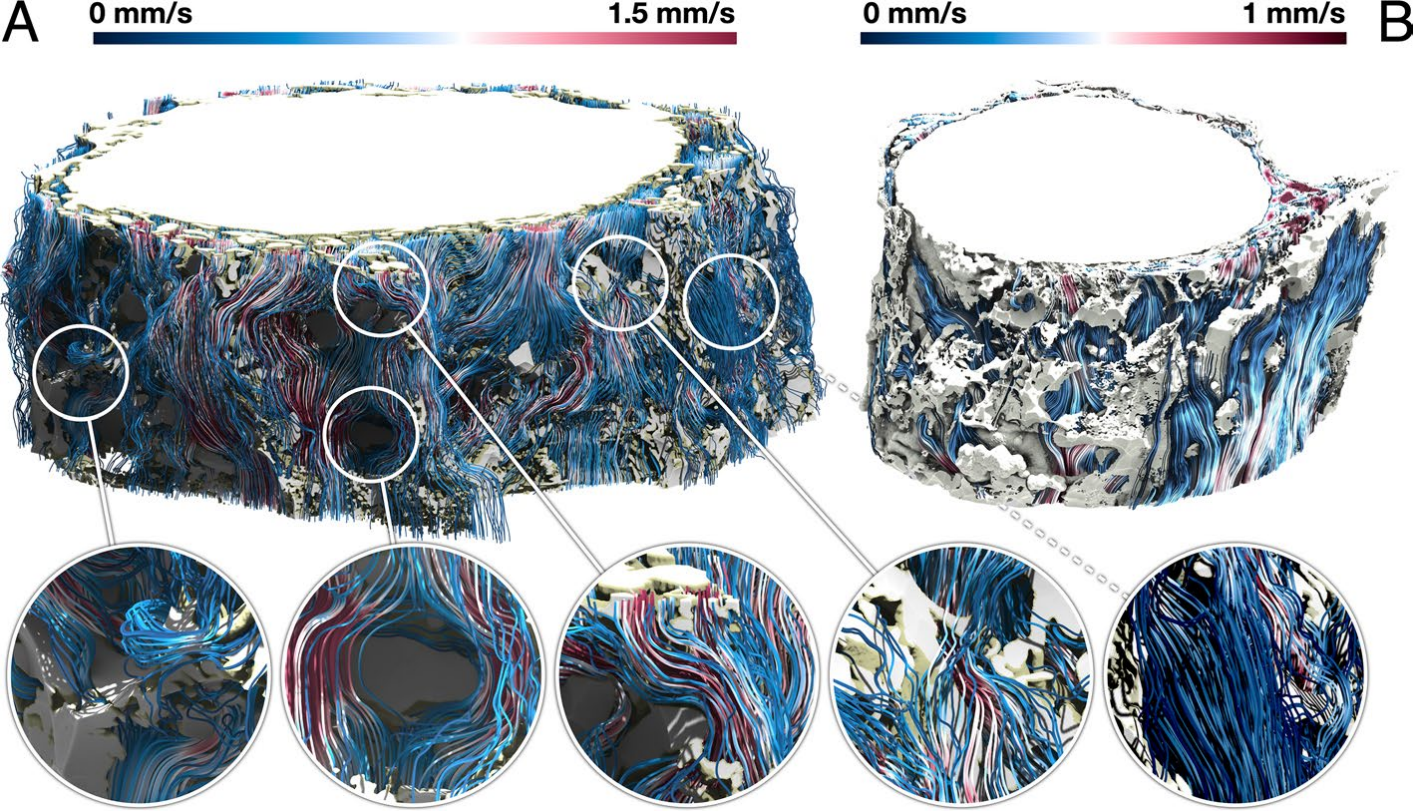
Streamlines of the CSF flow in *Dataset1* **A**, bulbar section) and *Dataset2* **B**, intraorbital section), coloured according to the flow speed, together with the ONSAS microstructure. Larger trabeculae have been removed for the sake of clarity

**Fig. 6:**
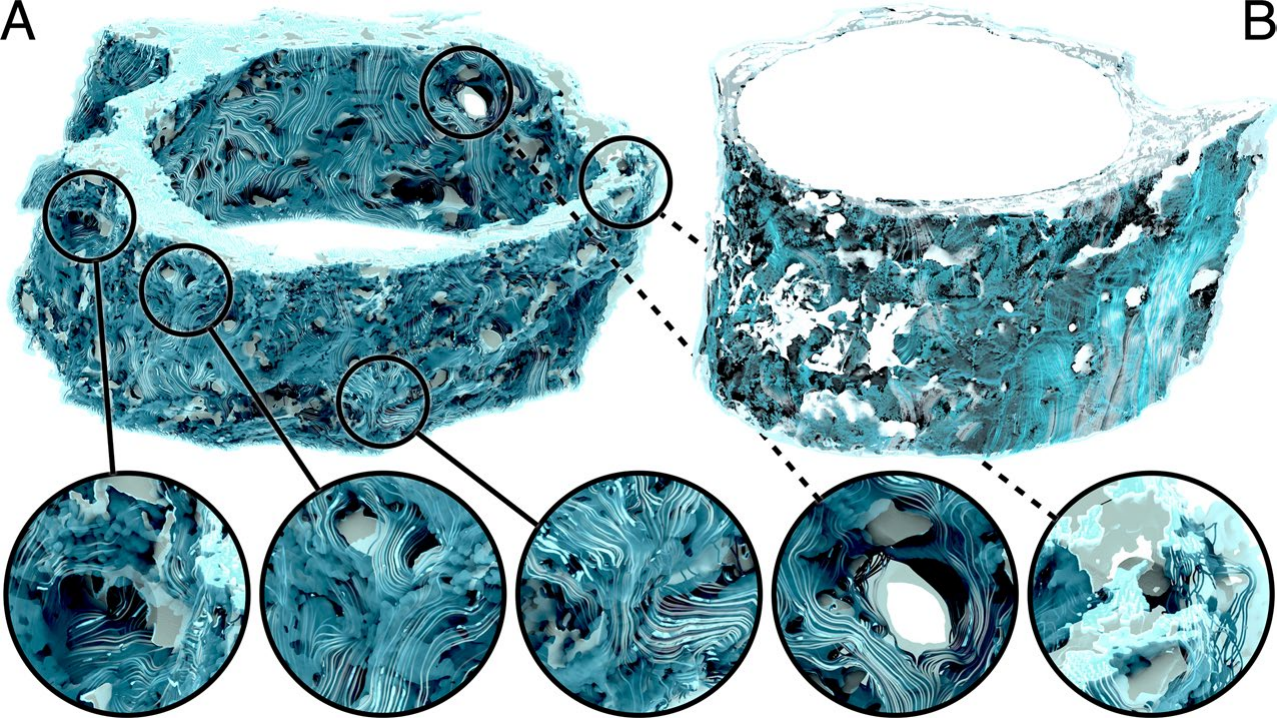
CSF-filled space (translucent green) and CSF flow (opaque streamlines), to better illustrate the tortuous flow is imposed by the highly heterogeneous features of the CSF-accessible space, both for *Dataset1* **A**, bulbar section, and *Dataset2* **B**, intraorbital section

Interestingly, the ONSAS microstructure exerts significant resistance against the CSF flow, forcing the latter to turn and bifurcate at different spatial scales. As expected from mass conservation, maximum flow speed is observed in constricted regions, as shown in Fig. 5. In Fig. 6, CSF space is displayed in relation to the flow, emphasizing the constraints that the CSF flow must follow.

**Fig. 7:**
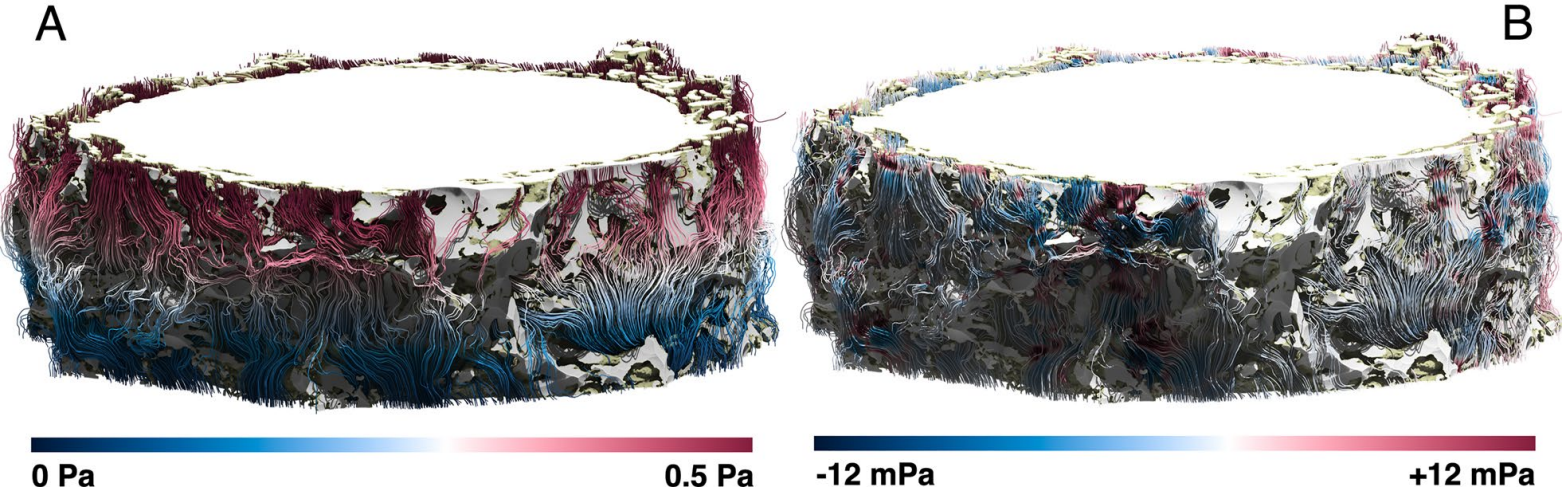
Streamlines of the CSF flow for *Dataset1* (bulbar section) coloured linearly according to the *total* pressure **A** and *dynamic* pressure **B**

In Stokes flows, the dynamic pressure is zero. Accordingly, we observe the total pressure of the CSF flow being dominated by the hydrostatic component. As shown in Fig. 7, the dynamic pressure (from -12 mPa to 12 mPa) appears to be substantially smaller than the prescribed hydrostatic pressure (500 mPa across the whole domain). Dynamic pressure is highest in the regions constricting the flow. As expected for low Re flow problems, symmetrical patterns appear upstream and downstream of narrowing spaces.

*Dataset2* was acquired from the intraorbital segment of the optic nerve specimen which exhibits a narrower ONSAS compared to *Dataset1* acquired from the bulbar segment. A visual inspection of the streamlines revealed that the *intraorbital section* is more prone to stagnation compared to* bulbar segments of the ON.*

**Fig. 8:**
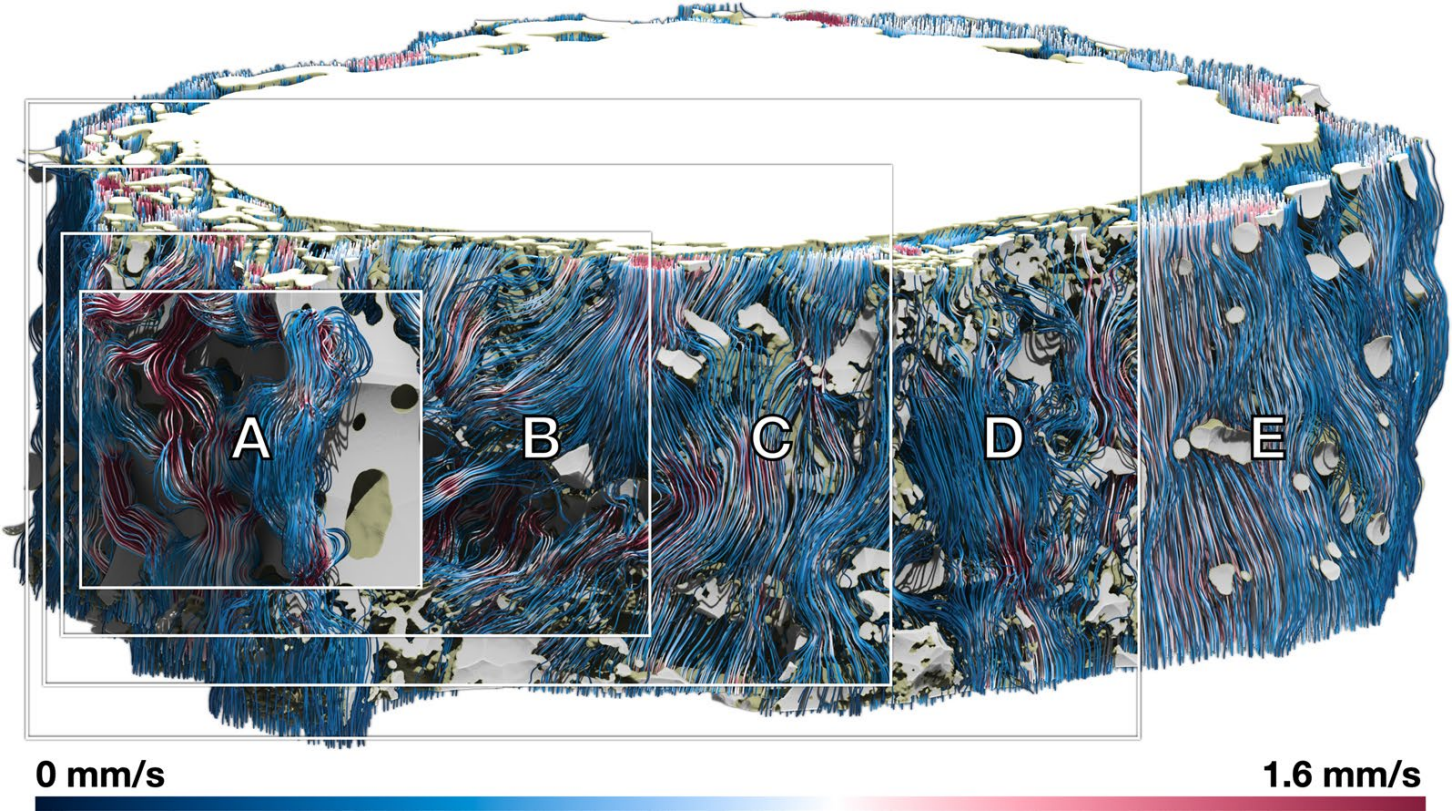
Streamlines of the CSF flow within *Dataset1* (bulbar section) for a morphological closing of 40 μm **A**, 10 μm **B**, unaltered geometry **C**, opening of 10 μm **D**, and opening of 50 μm **E**, respectively, coloured by flow speed when applying a constant pressure of 0.5 Pa across the domain

Figure 8 provides insight on how CSF flow changes in response to manipulation of the geometry. Closing operations lead to confined spaces achieving higher flow speeds and sudden turns, whereas opening operations make the flow stream more homogeneously through the ONSAS. We encourage the reader to consult the Additional files 4, 5, to gain a better insight about how the geometrical manipulations affect the CSF flow speed and pressure, respectively.

Accordingly, we calculated the pressure differential necessary to attain a physiological CSF flow speed (Table 3). Consistent with the narrower geometry, the necessary pressure differential in intraorbital ON segments is slightly higher compared to bulbar segments. A direct extrapolation suggests that a pressure differential of 15–27 Pa (0.11—0.20 mmHg) would be sufficient to maintain a CSF flow streaming on average at 0.5 mm/s of an average optic nerve length of 4 cm.

**Table 3:** Hydrostatic pressure differential required to match CSF flow speeds observed in human subjects

Dataset	Average speed	Re_D_ [−]	ROI “thickness”	Volumetric rate	ΔP
*Dataset1 bulbar s*	0.5 mm/s	0.1	0.75 mm	0.095 mL/min	0.28 Pa
*Dataset2 intraor. s*	0.5 mm/s	0.1	0.73 mm	0.074 mL/min	0.49 Pa

**Fig. 9:**
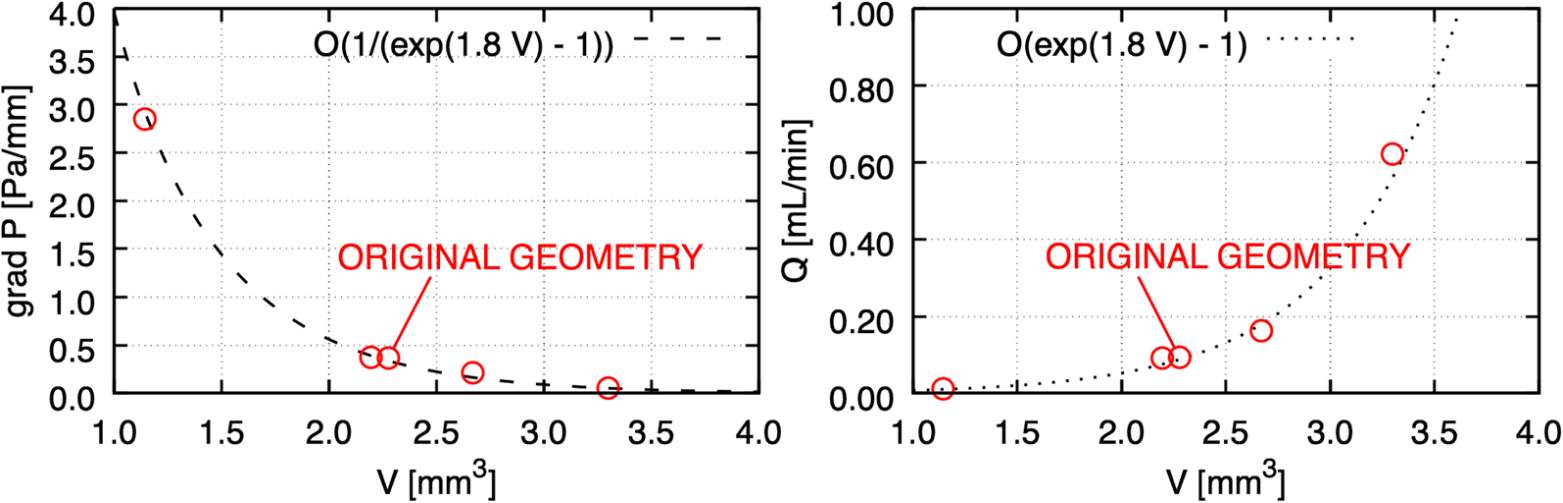
CSF flow changes in response to the CSF accessible volume of the manipulated geometries. Required pressure gradient to attain a CSF volumetric flow rate of 0.095 mL/min (left), and volumetric rate in response to a constant pressure gradient of 0.37 Pa/mm (right)

We explore the effect of altering the volume of CSF-accessible space and ONSAS architecture, e.g., as encountered during optic nerve compartment syndrome. As shown in Fig. 9 (left), the relationship between pressure gradient and volume seems well approximated by a curve featuring an exponential in its denominator, and a singularity at zero. A morphological opening on the trabeculae results in an increased volume accessible to CSF and leads to a quasi-linear relationship between volume and pressure gradient. In the limit case, a total morphological closing of the trabeculae is equivalent to a complete removal of CSF-accessible volume resulting in infinite pressure inside the structure. This follows directly from the no-through boundary condition preventing the CSF from streaming through the wall.

Figure 9 (right) is obtained by imposing a pressure gradient of 0.37 Pa/mm (taken from Table 3) on the geometry and measuring the response in terms of volumetric flow rate, by leveraging the direct proportionality between pressure drop and volumetric flow rate.

### Mass transfer

**Fig. 10:**
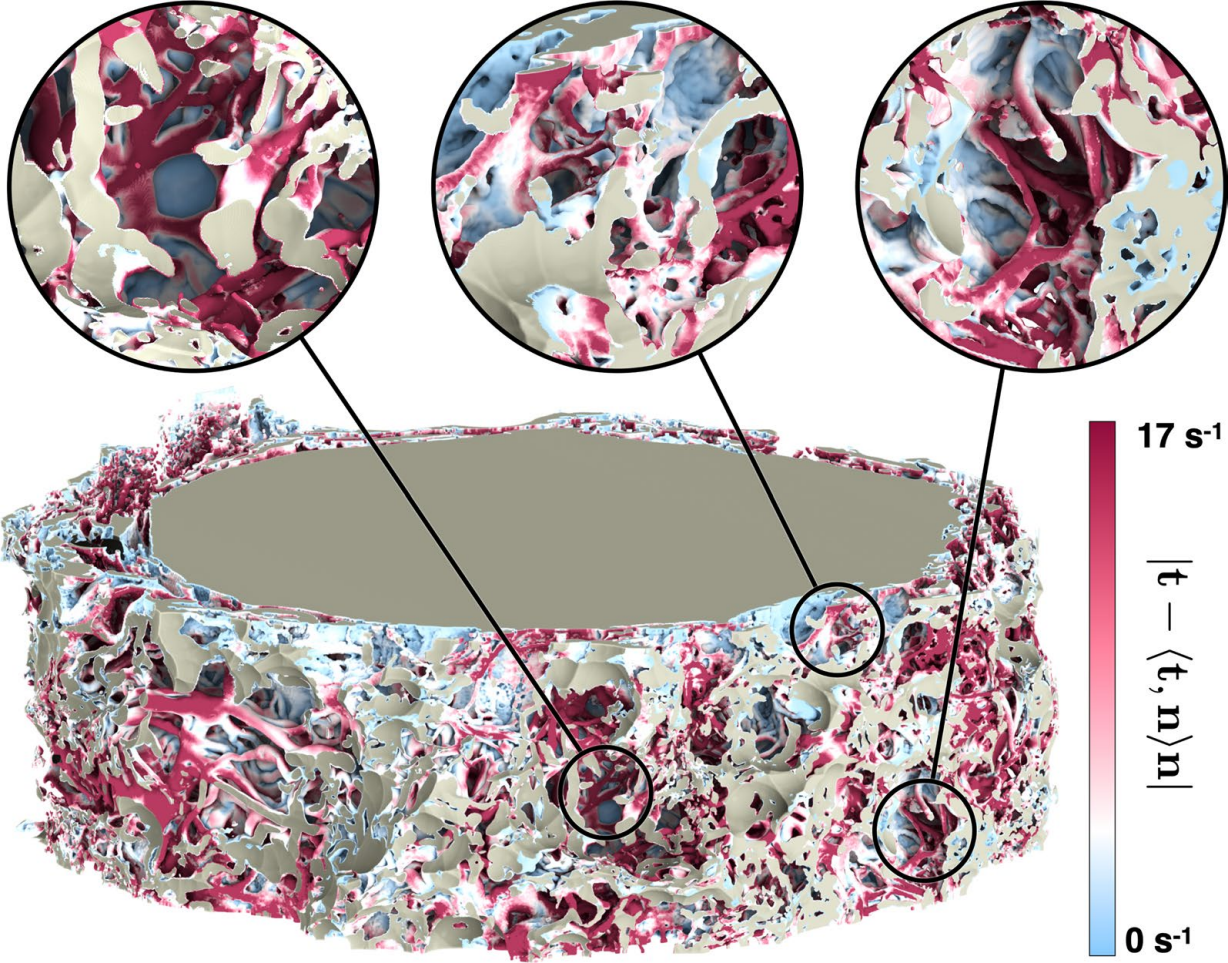
Visualization of the homeostasis proxy (strain rate magnitude tangential to the wall, expressed as ($\left| {t - \left\langle {t,n} \right\rangle n} \right|$) on the original ONSAS geometry (bottom) of *Dataset1* (orbital section). Well-separated trabeculae display consistently larger values compared to other structures, as emphasized in the close-ups (top)

To better understand the potential of various surface areas to contribute to material exchange with the CSF, we calculated the magnitude of the strain rate tangential to the wall (Fig. 10), the homeostasis proxy described earlier in the text. The prominent characteristic is its heterogeneity across the surface. However, the trabecular structures, especially those more apart from each other, consistently display a value higher than the rest of the ONSAS microstructure. This suggests that “well separated” trabeculae offer superior material transfer to the CSF flow and plausibly contribute more to homeostasis. A possible physical explanation is that isolated pillars lead to flow bifurcation around them, thereby increasing the strain rate at their surface, with the side effect of increasing overall drag exerted by the microstructure.

Alterations of the ONSAS geometry caused significant changes to the pressure differential necessary to drive CSF flow and associated volumetric flow. Therefore, we want to explore what effects these alterations have on potential mass transfer between CSF and meningeal surface.

**Fig. 11:**
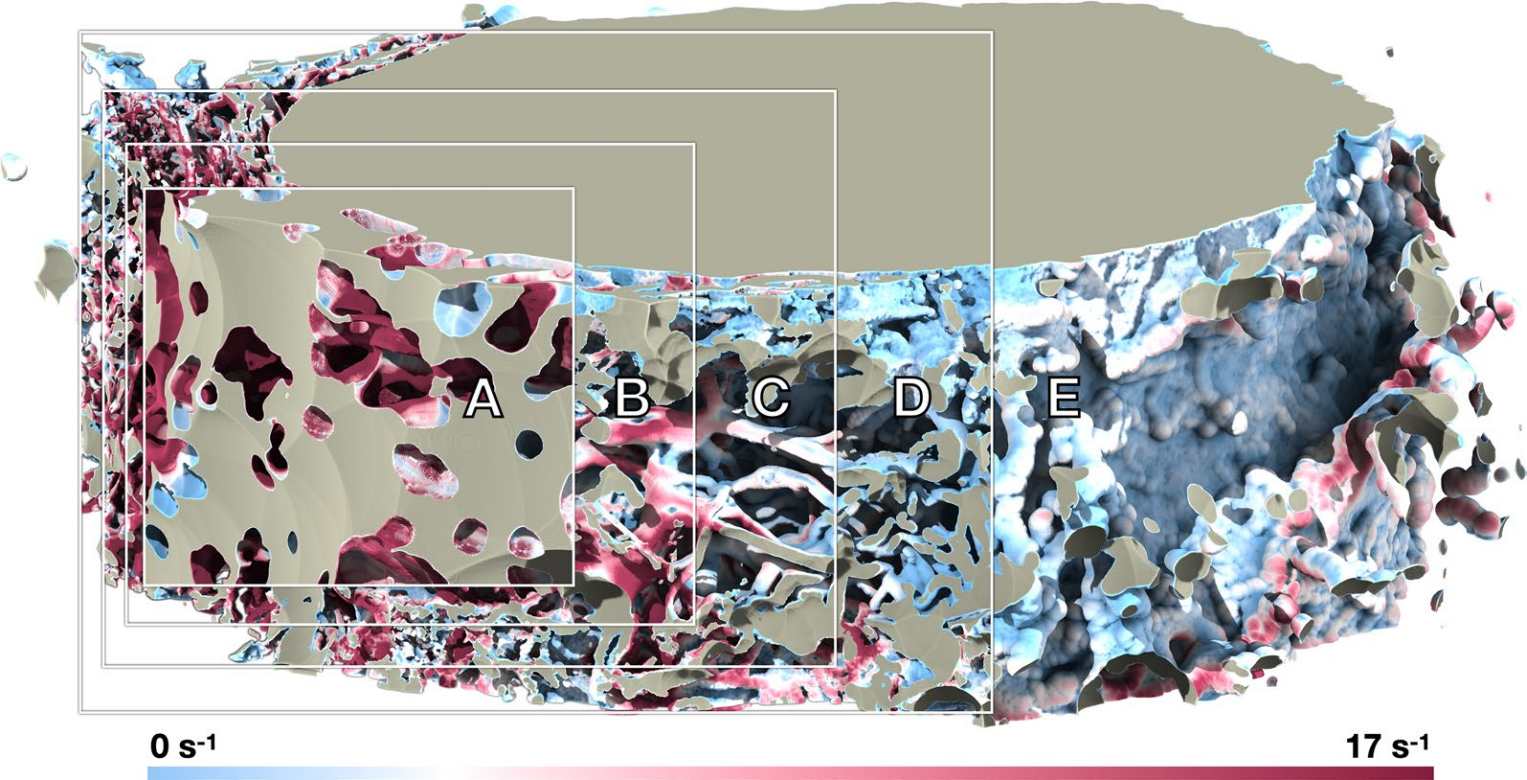
Wall shear strain magnitude of *Dataset1* (bulbar section) for a morphological closing of 40 μm **A**, 10 μm **B**, unaltered geometry **C**, opening of 10 μm **D**, and opening of 50 μm **E**, respectively, coloured by flow speed when applying a constant volumetric rate of 0.095 mL/min through the ONSAS

Figure 11 reveals how the geometrical manipulation influences the strain rate magnitude at the wall, for a prescribed volumetric rate. Closing operations removes CSF-accessible volume, and thus lead higher speed while exposing smaller cross-sectional area, whereas opening operations significantly increase the volume, thus widening the CSF cross-sectional areas and forcing the flow to slow down to maintain the prescribed volumetric rate. The absence of trabeculae causes a large decrease in mass transfer.

**Fig. 12:**
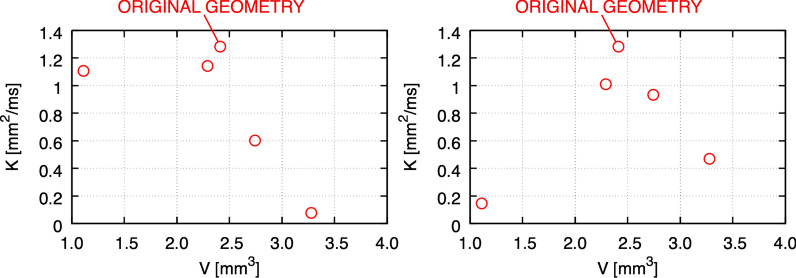
Mass transfer capability in relation to the volume of the geometrical manipulations, at constant volumetric rate of 0.095 mL/min (left) and under a constant hydrostatic pressure gradient of 0.38 Pa/mm (right), for *Dataset1* (orbital section)

**Fig. 13:**
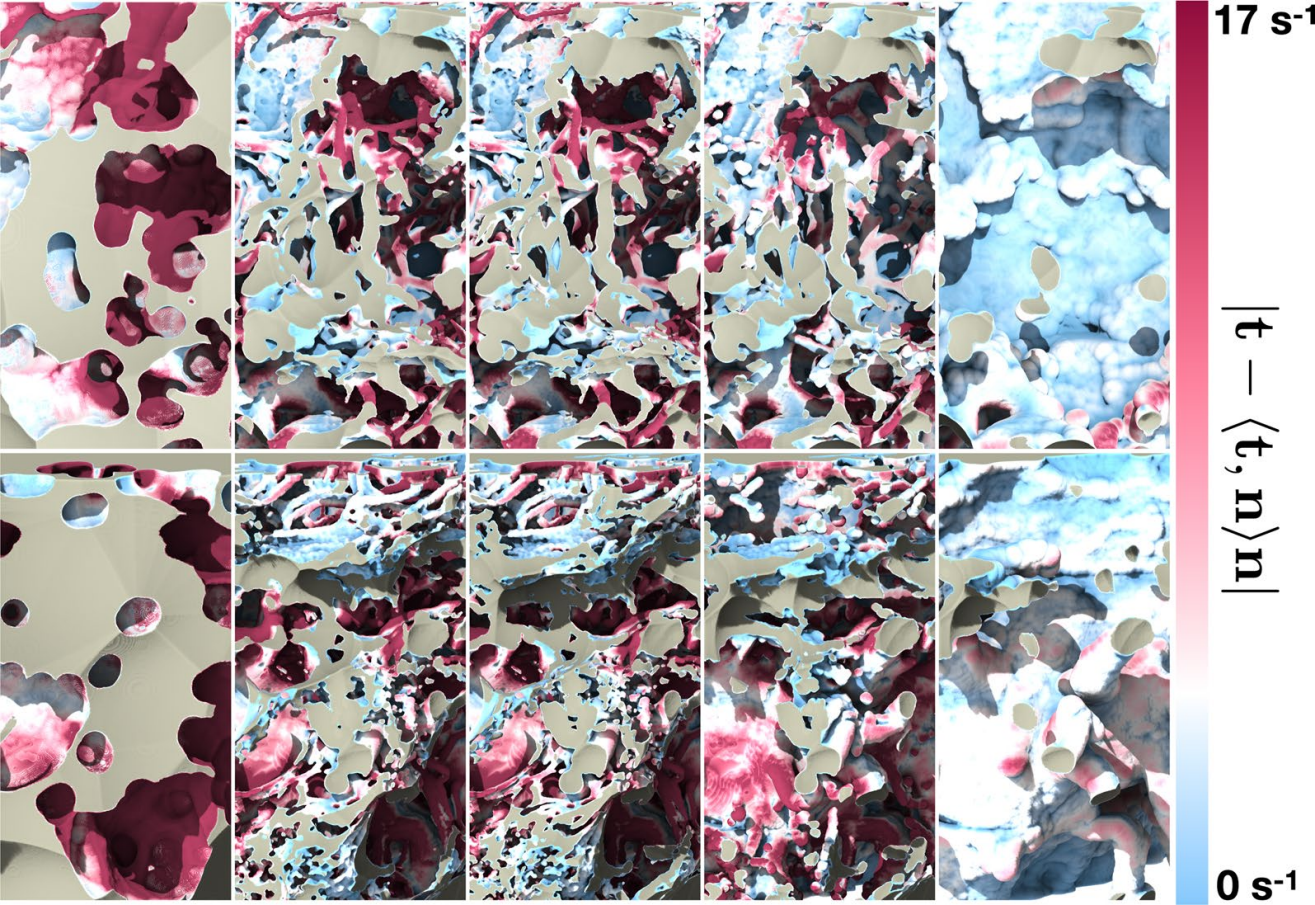
Mass transfer on the examined geometrical configurations of *Dataset1* (orbital section), sorted with respect to the strength of the applied morphological manipulations (from left to right: closing, original and opening of the microstructure), at constant volumetric rate of the CSF flow, showing how the progressive removal of the structure may affect homeostasis

Figure 12 summarizes the overall mass transfer versus the volume of the geometrical configurations, when maintaining the prescribed volumetric rate (left), or under the prescribed pressure gradient (right). The two plots are remarkably different. This is somewhat surprising, as in a Poiseuille flow within a circular pipe one expects the same response by controlling either pressure gradient or volumetric flow rate. In turn, the plots provide evidence about the crucial role played by the ONSAS microstructure morphology. The original geometry demonstrates superior mass transfer capability in both conditions. Under constant pressure gradient, the relationship between *K* and volume is resemblant of the relationship between area and volume of Fig. 3. The left plot of Fig. 3, however, shows that a morphological closing on the ONSAS microstructure leads to slightly inferior mass transfers, and a strong morphological opening would lead to almost no mass transfer. Figure 13 provides details on how the wall strain rate responds to the geometrical manipulations, on two distinct regions of interest in *Dataset1*. Readers are encouraged to consult Additional file 6 to gain deeper insight about how the wall strain rate is affected by the geometrical configurations.

### Reduced Order Models and Idealized Geometries

**Fig. 14:**
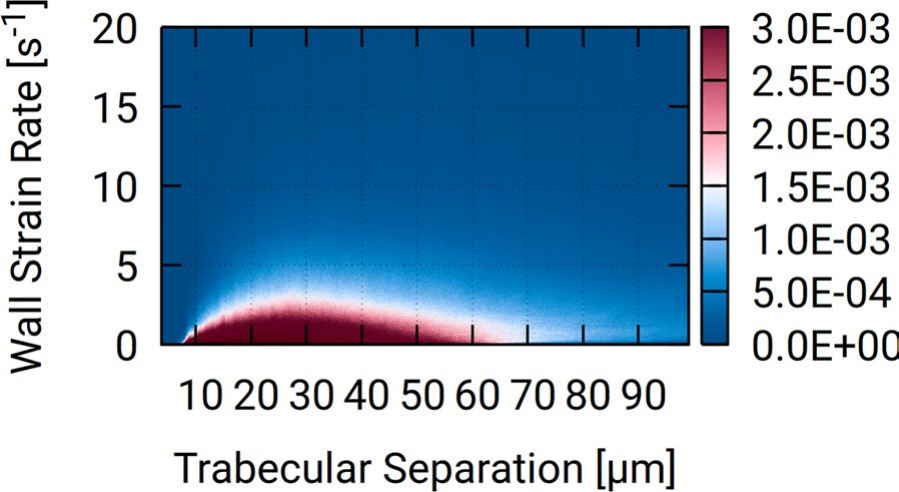
Adaptive histogram approximating the probability density function between trabecular separation with strain rate at the wall, for *Dataset1* (orbital section)

We wish to investigate whether there is a clear relationship between purely geometrical features and its CSF dynamics, in order to avoid expensive use of DNS. Figure 14 depicts the histogram of CSF space thickness (i.e. trabecular separation, as computed in [20], and wall strain rate. Within CSF-accessible pockets, trabecular separation is large, but the flow speed slows down due to mass conservation leading to small strain rate. Conversely, trabecular separation is small within narrow sheet-like spaces where indeed the flow is almost stagnating and exhibiting low strain rate. The fact that wall strain rate can be low in both cases (high and low separations) prevents a straightforward approximation of the former quantity. Similar difficulties are encountered in Earth Science where geometrical parameters of tortuosity fail to predict transport properties of physical tortuosity [49]. The seemingly common difficulty of capturing correlations between geometrical descriptions and physical properties perhaps advocates the use of first-principle calculations rather than adopting reduced-order modeling whenever possible.

**Fig. 15:**
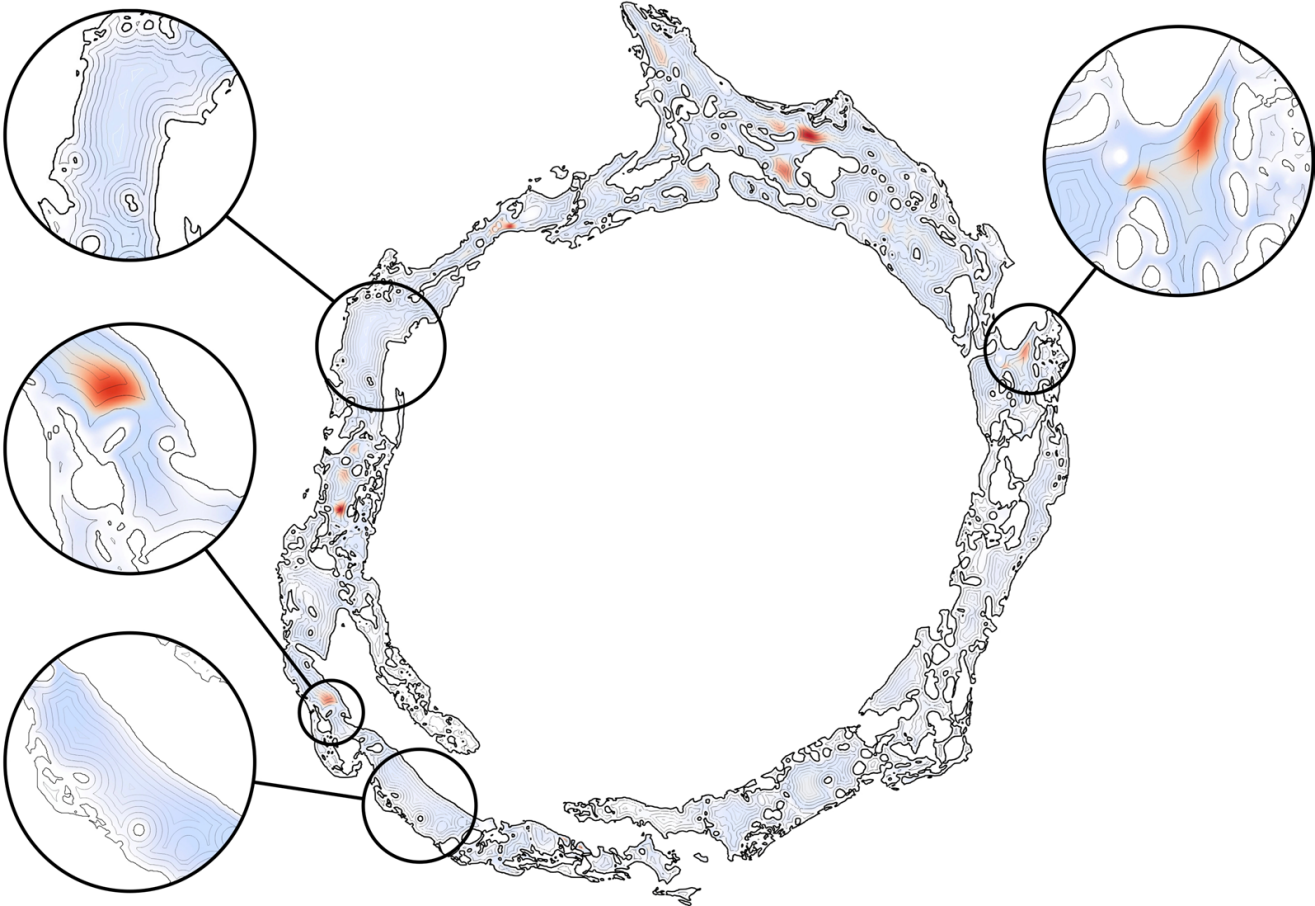
CSF flow speed (blue/red denotes low/high CSF flow speed) overlayed by the isocontours of the 3D Euclidean distance from the wall, highlighting the rather surprising absence or location of peaks with respect to where one may expect them

Based on the knowledge gathered from flows on idealized geometries, one may expect flow speed peaks at locations that are farthest away from the wall boundaries. Figure 15 depicts the relationship between the CSF flow speed and the 3D Euclidean distance from the meningeal layers. The figure invalidates such expectation and shows that presence/absence and location of peaks does not obey any simple rule. This may suggest that CFD investigations on idealized geometries may have limited applicability and relevance to CSF dynamics in real anatomical structures.

## Discussion

We present an *in-silico* large-scale investigation in the subarachnoid space of the optic nerve at a hitherto not available level of detail, to study CSF dynamics under normal and potentially pathological conditions. The calculations show that CSF does stream heterogeneously through the ONSAS and that different flow patterns can be observed at different locations (as shown in Fig. 5). The examined ONSAS geometries force the flow to change direction and bifurcate. As such, they can lead to significant resistance to the CSF flow. Despite this fact, the resulting pressure drops are negligible compared to the normal values of ICP (i.e., 10-15* mmHg*): if we extrapolate the pressure loss throughout the optic nerve we get an overall drop of 0.1–0.2 mmHg, assuming that the ON length is about 40 mm. In summary, the observed results agree with the hypothesis that the ICP is transmitted without significant losses to the CSFP in the retrobulbar region, under normal conditions and with the unaltered ONSAS microstructure. The dependency between ONSAS geometry and CSFPG has been examined.

Despite the relatively low pressure drop required to drive the CSF flow at the target velocity, we have observed that some regions are substantially exposed to flow stagnation, and obtained visual confirmation of strong local heterogeneity in flow speed across the ONSAS. This may relate to pathophysiological conditions such as glaucoma [38]. Such regions seem more prominent in the intraorbital region more than in the retrobulbar region. This seems in contrast with the experimental finding of tracers accumulating in the retrobulbar region [38]. However, due to incompressibility, the intraorbital section is expected to slow down the CSF flow across the whole ONSAS, allowing for minimal dispersion at the retrobulbar region. This could be investigated numerically in a follow-up paper, by applying the same approach presented herein.

A quantification of the relationship between ONSAS geometrical details and CSF flow is valuable for several reasons. Firstly, it serves as a sensitivity analysis propagating the uncertainty in the image segmentation to computational modeling. Secondly, it mimics the morphological changes symptomatic of certain pathophysiological processes. Altered anatomical conditions for example are the formation of cell nests that are likely to impact flow conditions as demonstrated by Pache et al. [40]. In addition, an in vitro study [2] of meningeal cells has shown an increased proliferation of these cells under elevated hydrostatic pressure. Morphological operations provide us with a systematic and well-controlled manipulation of the ONSAS microstructure and have been also extensively employed to create the underlying image segments [20].

When the ONSAS microstructure is made less voluminous, the relationship between volume gain versus necessary pressure drop appears to be quasi-linear. When the ONSAS microstructure is made thicker, the relationship is highly nonlinear, leading to a diverging pressure in the limit case of a collapsing ONSAS. Both behaviors are well approximated with a straightforward expression with the exponential function. In a limit case scenario of severe obstruction, the ONSAS microstructure requires a pressure gradient of 2.84 Pa/mm to attain a CSF rate of about 0.1 mL/min, amounting to a pressure drop of 0.85 mmHg over 4 cm.

The link between homeostasis and ONSAS geometry has been examined quantitatively in this study. ONSAS trabeculae, especially those ones that are well separated, are probably key contributors to homeostasis. We leveraged boundary layer analogies to formulate a global metric to compare mass transfer properties of different geometries, based on the component of the strain rate tensor tangent to the wall, from the CSF flow field approximated by DNS. The numerical investigation suggests that the unaltered ONSAS geometry is robustly optimized towards homeostasis, compared to the morphologically altered configurations. Figure 13 may provide a clue about it: a weak closing operation on the structure leads to a qualitatively similar geometry, although with a slight decrease in area. The decrease in area is partially compensated by a higher flow speed due to a smaller cross-sectional area. The end effect is a slight decrease of the overall mass transfer. An opening operation, on the other hand, tends to decrease surface area as well, but substantially decrease flow speed by increasing cross-sectional area. In addition, an opening tends to break apart or completely remove the trabeculae. As mentioned earlier, the latter are likely to be key contributors of mass transfer.

While a straightforward surface area comparison between the original geometry and one without microstructure leads to a ratio of 3:1, comparable to previous findings [20], the ratio of the homeostasis capability according to the presented metric is 17:1, for an equal volumetric rate.

### Relevance of the present investigation

Disturbed CSF composition and dynamics is thought to be involved in neurodegenerative pathologies such as Alzheimer’s disease, normal tension hydrocephalus, Parkinson’s disease and IIH. Within the ON, elevated CSF pressure causes stasis of axoplasmic flow that results in papilledema [14]. The latter is a hallmark of elevated ICP such as IIH, and intracranial mass lesions. ICP has also gained interest in the pathophysiology of normal tension glaucoma (NTG). Some studies found lower ICP in NTG patients compared to healthy individuals. It is hypothesized that the pressure gradient across the lamina cribrosa tends to deform the lamina cribrosa towards the optic nerve, therefore causing shearing forces within the lamina that results in axon loss and optic disc cupping [11]. CSF flow studies applying computed tomography assisted cisternography and diffusion weighted magnetic resonance imaging demonstrated reduced contrast loaded CSF within the SAS of the ON in a cohort of patients with IIH and NTG. Flow ratios within the ONSAS were reduced in IIH patients when compared to age compared controls [6]. Both results were compatible with a disturbance of CSF dynamics in the ONSAS.

Although pressure related damage to optic nerve axons is the generally accepted mechanism for the development of papilledema, CSF stagnation is another possible mechanism contributing to axonal injuries. As CSF acts as a transport system for neurotransmitters, proteins, peptides, minerals, and metabolic waste products to and from the brain, CSF dynamics has therefore a strong influence on the homeostasis of the microenvironment of the brain and optic nerve. Lack of CSF clearance in a compartmentalized ONSAS has been hypothesized to contribute to axonal damage in papilledema caused by IIH. Lack of CSF clearance is thought to result in the deposition of neuronal waste products within the brain parenchyma and axons. “Toxic” CSF has recently been linked to frontotemporal dementia and to amyotrophic lateral sclerosis [32].

### Towards Exascale Investigations

The majority of the computational studies on CSF dynamics carried out so far relied on drastic simplification of the geometry, or reduced order model. As reported in Fig. 14, a consistent approximation of the CSF dynamics by considering exclusively geometrical features does not seem possible, due to the non-local nature of hydrodynamic interactions. Results depicted by Fig. 15 indicate that accuracy –hence physiological relevance– of studies on idealized geometry may be severely limited in their relevance, as detailed geometry combined with detailed first-principle flow mechanics (DNS of NSE) seem to constitute a building block of CSF-mediated homeostasis.

This motivates the need of carrying out further large-scale CFD investigations within the ONSAS. An exascale analysis of the full optic nerve would be valuable for different reasons. Firstly, geometrical patterns are heterogeneous across the optic nerve. Their flow interaction is expected to lead to long-range effects due to pressure and viscous forces (low Reynold numbers). Such study would be globally consistent and at a resolution enough to carry out detailed analysis of all sections of the optic nerve, including the intracanalicular section. Secondly, modeling the entire optic nerve would allow the examination of how CSF enters and moves from the intracranial space into the optic nerve ONSAS, possibly revealing universal driving mechanisms of the CSF [30]. Thirdly, a study on the full ONSAS is set to shed light about mechanistic details, e.g. why the retrobulbar region seems clinically more exposed to stagnation whereas numerically the CSF in the intraorbital section seems more vulnerable.

### Limitations of the present study

We acknowledge several limitations of this study. Firstly, the subarachnoid space of the optic nerve does not only contain trabeculae but also septae that display a different geometry. This work is focused on the anatomy and geometry of the trabeculae in two whole slices of the ON. Therefore, the study is not completely representative of the SAS of the ON. Future work may include the analysis of whole slices from other sections, which were not present in the considered specimen.

A second limitation is given by the assumptions of the computational model. The exact boundary conditions at the meningeal layer are unknown. Between a fluid flow and a bounding surface, partial slip is expected when fluid is a water-like liquid and the surface is coated with a lipid bilayer, due to electrostatic forces. This presumably is the case for the meningeal layer and CSF. However, by imposing a no-slip condition we are estimating an upper bound on the needed pressure differential.

The no-through condition is ensuring that pressure is spent only to drive the flow through the ONSAS. On the other hand, to maintain the target flow rate at the outlet, the required pressure difference inevitably must increase, if the structure is leaky or porous, compared to the same geometry built with an impermeable material.

Furthermore, the two specimens were imaged ex vivo and the lack of physiological conditions such as blood pressure, respiration, and heart rate may represent significant limitations. Our approach assumes that ambient pressure and fluid temperature are constant and homogeneous, and thus do not affect the flow field. We believe that blood pressure and body temperature do not necessarily affect the results reported herein.

Moreover, the present approach ignores the pulsatile patterns of ICP and CSF flow. In the present work, the Reynolds number is computed directly from spatiotemporal averages acquired experimentally from MRI [15]. In turn, our results refer to mean flow quantities. For a pulsatile flow reaching higher speeds, however, up about Re ~ 10, transiency is not expected to influence the spatial flow pattern, as there is a clear separation within the spatiotemporal scales. We refer to [33] for a detailed explanation of the underlying mechanism.

A third limitation is that the present computational model did not include structural deforming effects due to the compliance of ONSAS. When CSF streams through the trabeculae, it exerts a mechanical load that may deform them significantly, altering the flow patterns during a pulsation. These effects have been reported to be relevant in relation to the spinal SAS [44]. Moreover, the bending of the ON during eye movements should be considered in further studies. Despite significant uncertainties regarding the material parameters, future work may consider reliable computational models of active and passive deformations. Furthermore, the optic nerve volume may decrease in glaucoma [34–36]. Another study reported that the orbital opening area in NTG patients is smaller than in healthy subjects [37]. Such findings suggest that structural forces within the ONSAS are very relevant from a pathophysiological perspective, and extending our analysis with two-way flow structure interaction models may improve the understanding of such conditions. Moreover, a recent study by Wang et al. [38] showed that fluid transport within the perivascular space is affected by aging and glaucomatous damage. Since the imaged tissues include the optic nerve parenchyma, we may assign porosity to our CFD model and assess its effect on the flow field. The immersed boundary schemes of our approach should facilitate such analysis.

A fourth limitation of the study is related to the possible morphological changes triggered by aging and/or the fixation procedure. The geometrical features of the ON specimen under examination may substantially differ from the ones of a younger subject. Furthermore, according to previous histological studies, inter-individual variability exists in the number and shape of trabeculae and septae. General statements on morphology can only be achieved by considering specimens from multiple donors. Moreover, the pathophysiological realism attainable by morphological operations further limits the predictive abilities of the proposed models. A follow-up work aims at extending the analysis of the flow response to geometrical manipulations by including soft tissue morphogenesis and growth/regression in the surface normal direction. Furthermore, morphological operations may be used to increase/decrease volume but inevitably decrease surface area. This further limits the generality of our finding when we compare the original geometry to the manipulated configurations.

Moreover, effects of postural position and the lack of CSF within the ONSAS, remain to be addressed. Compared to experimentation, a key advantage provided by in-silico analysis is that it scales out formidably well. Future investigations could evaluate the flow response to different displacements of the ONSAS for standing up or lying down positions.

### Additional considerations

In addition to diffusion-weighted MRI and CT-assisted cisternography, we envision the possibility of more recent in vivo imaging approaches [39] to validate the in-silico analyses under physiological conditions. Moreover, clinical experience seems to suggest that the most vulnerable section is directly behind the lamina cribrosa, in the bulbar segment. By carrying out a follow-up study with dedicated computational resources, in future we could apply the same morphological study on *Dataset2* as we did on *Dataset1* and make a quantitative comparison in this direction.

## Conclusions

Simulations suggest that the pressure drop of 0.1–0.2 mmHg over 4 cm seems sufficient to steadily drive CSF through the entire subarachnoid space, indicating low hydraulic resistance. Great heterogeneity in flow speeds, however, puts certain areas of the ONSAS at stagnation risk. A clear dependency between ONSAS geometry and CSF flow is revealed when manipulating the original geometry with morphological operations. Compared to the altered geometries, the original ONSAS architecture seems optimized towards maximization of homeostasis across a wide range of pressure gradients and volumetric rates. The homeostasis might be primarily carried out by the ONSAS trabeculae. These observations might shed light on pathophysiological processes leading to damage associated with insufficient CSF flow and optic nerve compartment syndrome.

### Supplementary Information

**Additional file 1: **Relationship between area and volume of different morphological operations.

**Additional file 2: **Overview of the manipulated geometry.

**Additional file 3: **Validation & Verification of the CFD approach.

**Additional file 4: **Streamlines of manipulated *Dataset1 *coloured according to flow speed.

**Additional file 5: **Streamlines of manipulated *Dataset1 *coloured according to dynamic pressure.

**Additional file 6: **Detailed visualizations of the wall strain rate of each of the geometrical configurations.

**Additional file 7: **Grayscale signal, and streamlines relating to the grayscale signal of *Dataset1*.

## Data Availability

Data relative to the present document will be made available on request. Please contact the corresponding author.

## Abbreviations

ON: Optic nerve
ON SAS: Subarachnoid space of the optic nerve
CSF: Cerebrospinal fluid
CSFP: CSF pressure
CSFPG: CSFP gradient
ICP: Intracranial pressure
