# Supplementary figures and images for: Large-scale in-silico analysis of CSF dynamics within the subarachnoid space of the optic nerve
Source: Fluids Barriers CNS. 2024 Feb 28;21:20. doi: 10.1186/s12987-024-00518-8 (PMC10900650; doi:10.1186/s12987-024-00518-8)

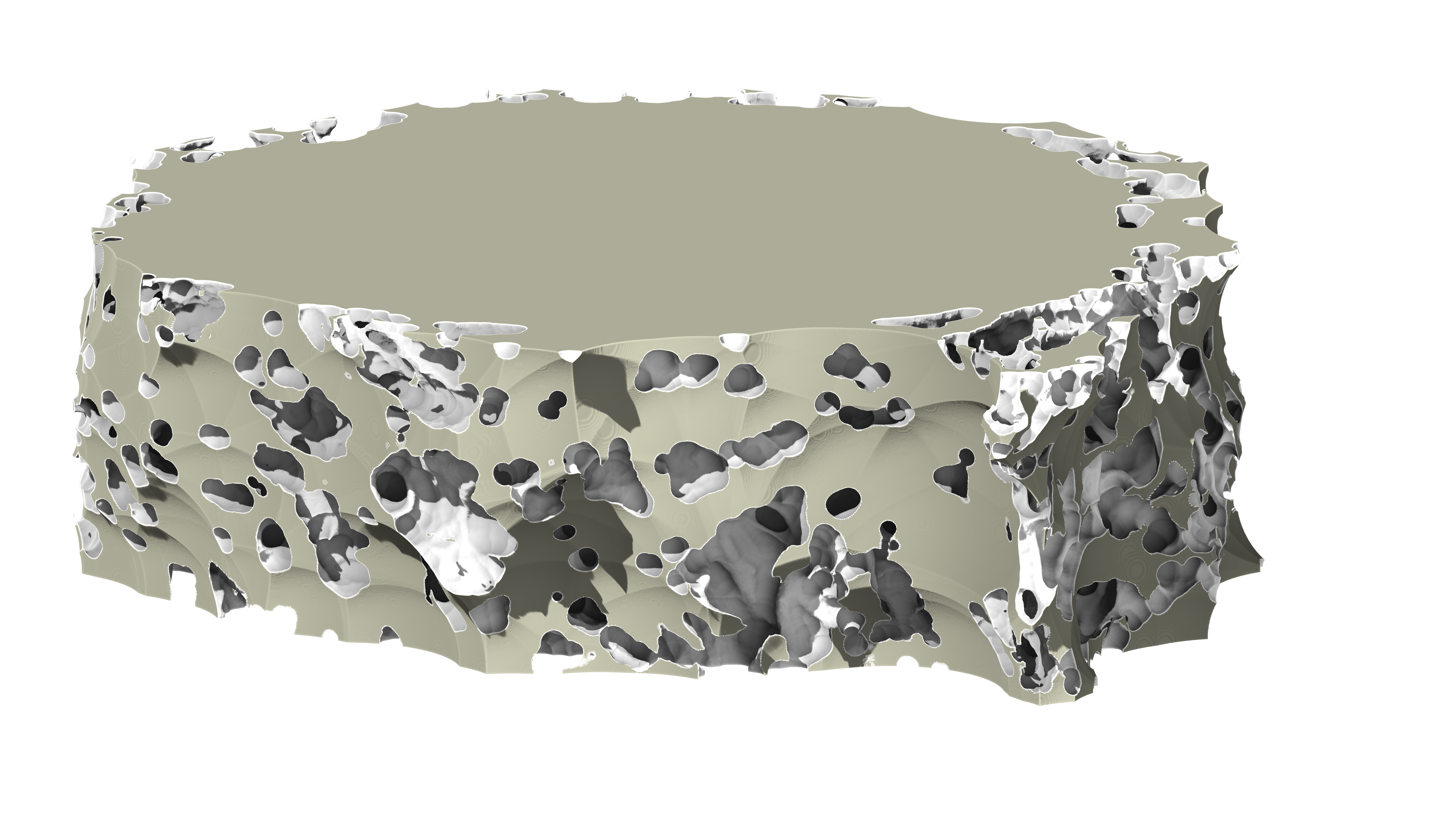

Supplement: Supplementary file 2 — Additional file 2: Overview of the manipulated geometry. [file 12987_2024_518_MOESM2_ESM.zip › S2/5_constricted.png]

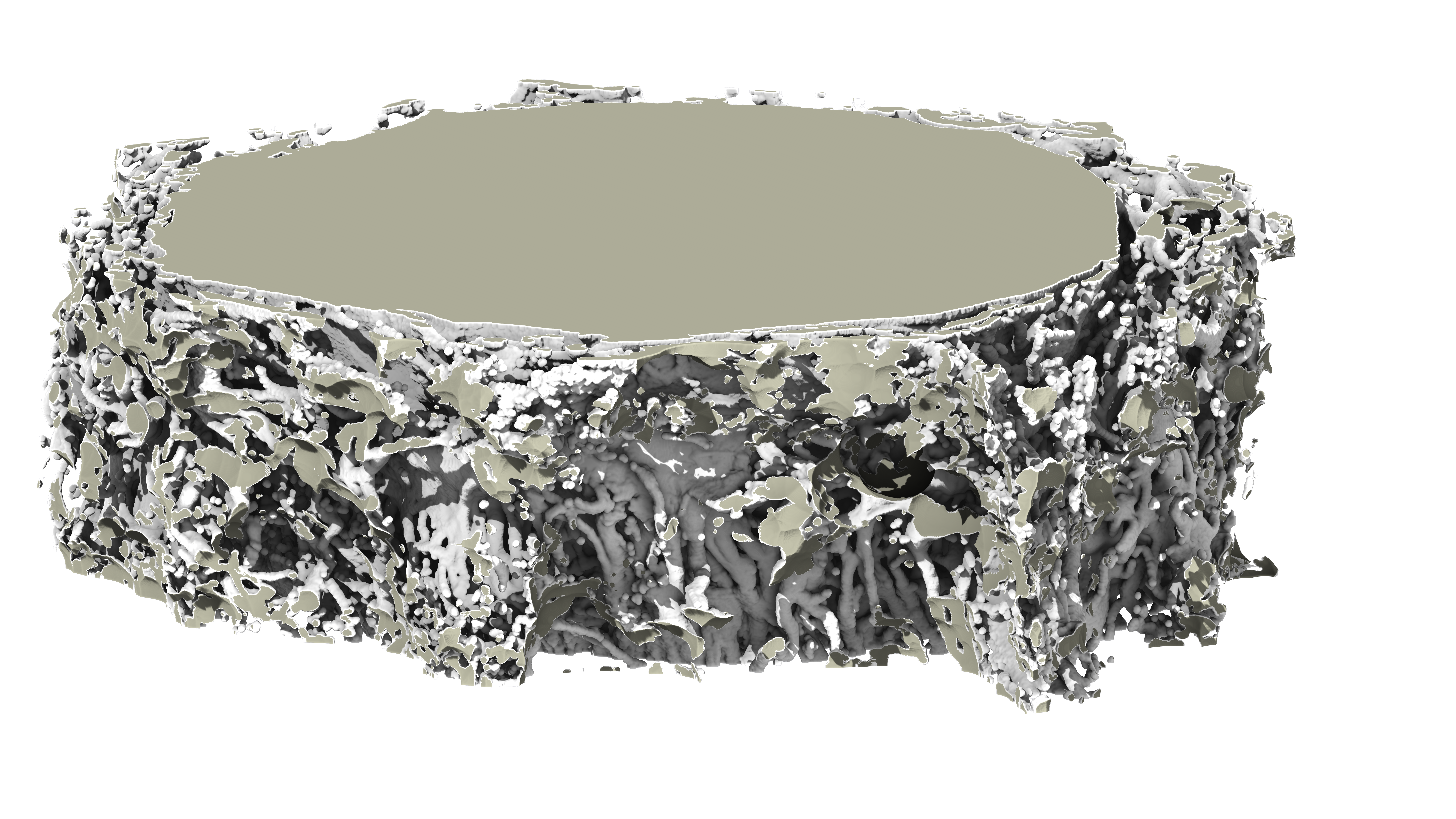

Supplement: Supplementary file 2 — Additional file 2: Overview of the manipulated geometry. [file 12987_2024_518_MOESM2_ESM.zip › S2/2_thinner.png]

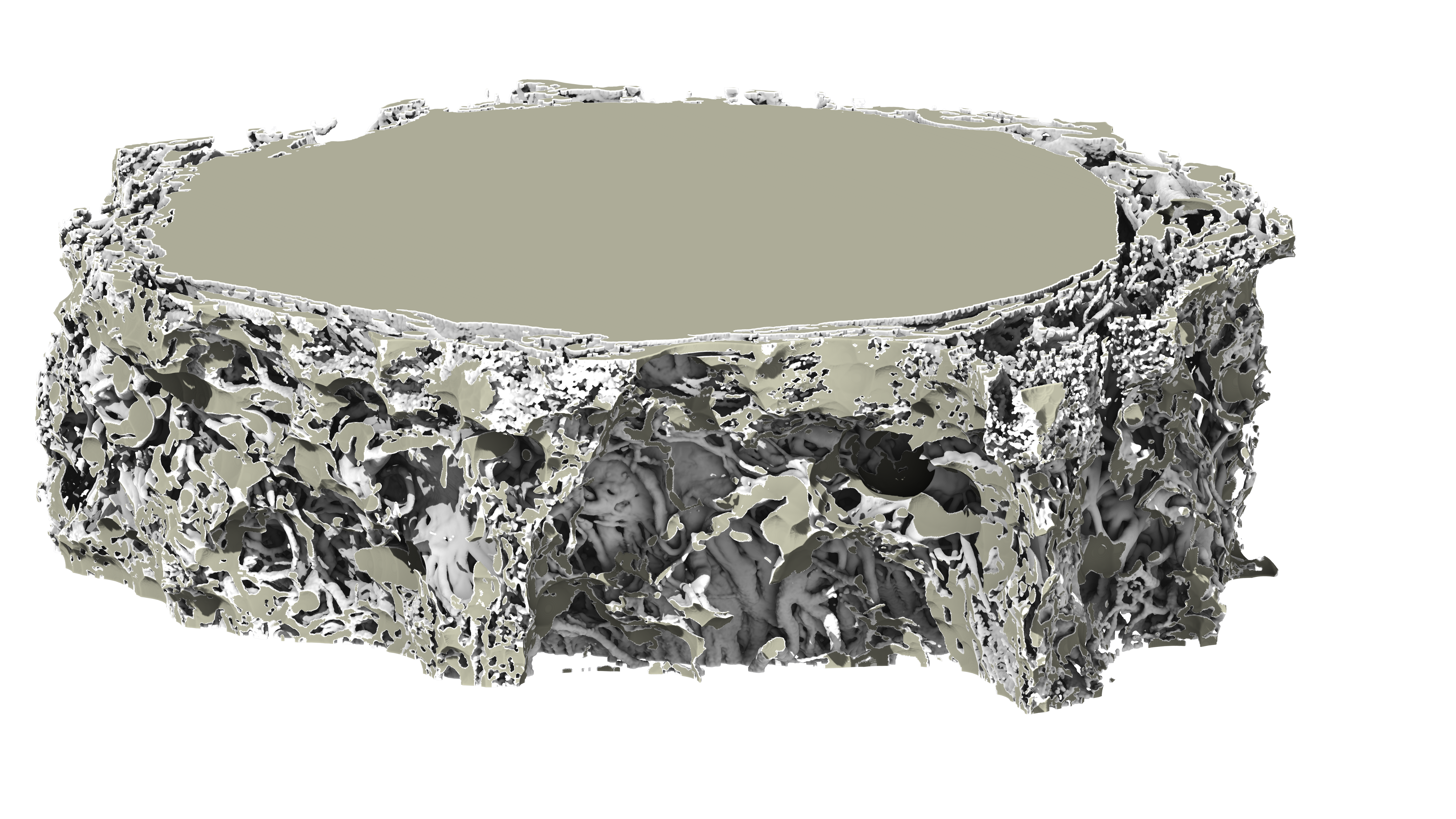

Supplement: Supplementary file 2 — Additional file 2: Overview of the manipulated geometry. [file 12987_2024_518_MOESM2_ESM.zip › S2/3_orig.png]

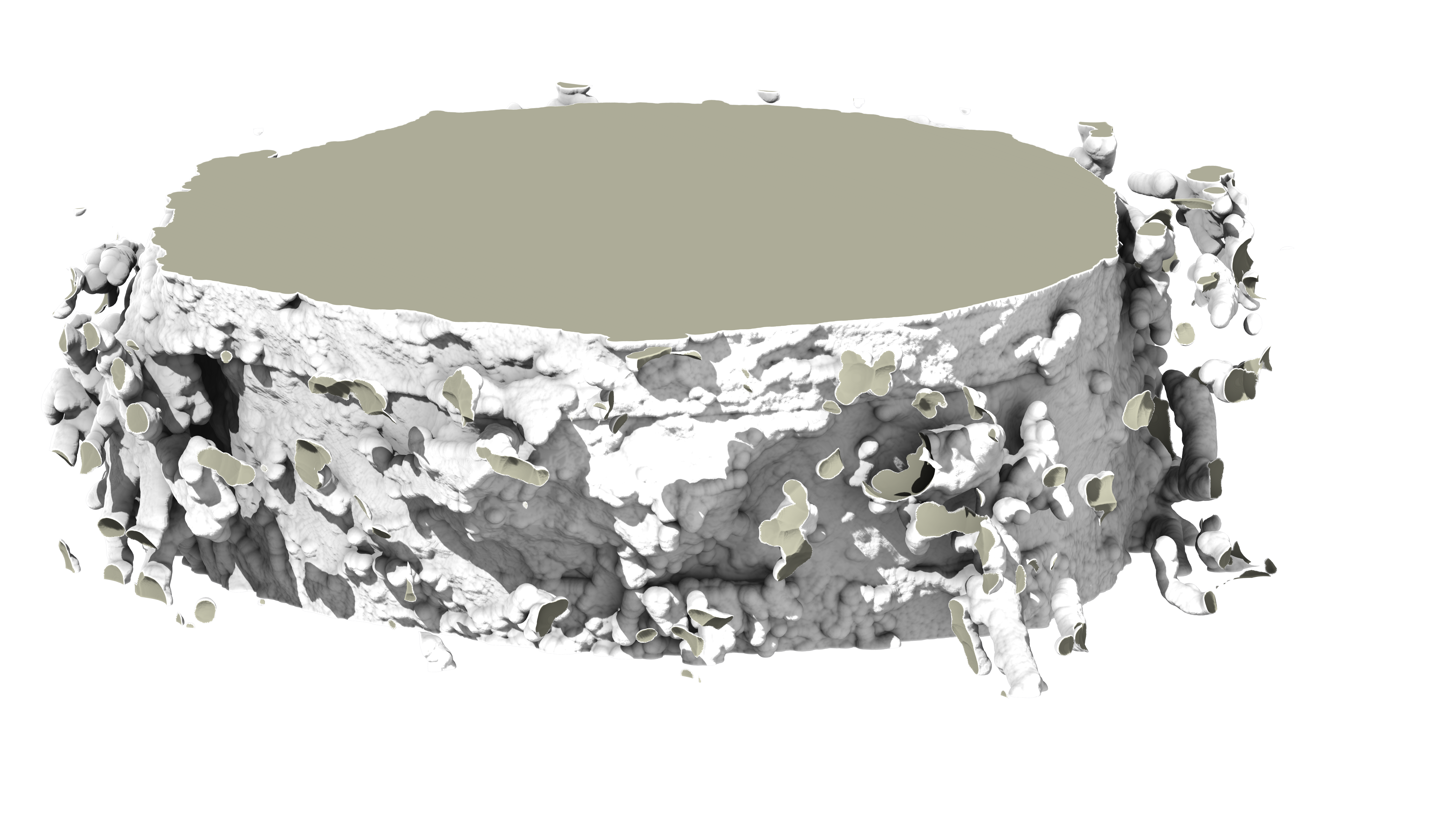

Supplement: Supplementary file 2 — Additional file 2: Overview of the manipulated geometry. [file 12987_2024_518_MOESM2_ESM.zip › S2/1_nouarch.png]

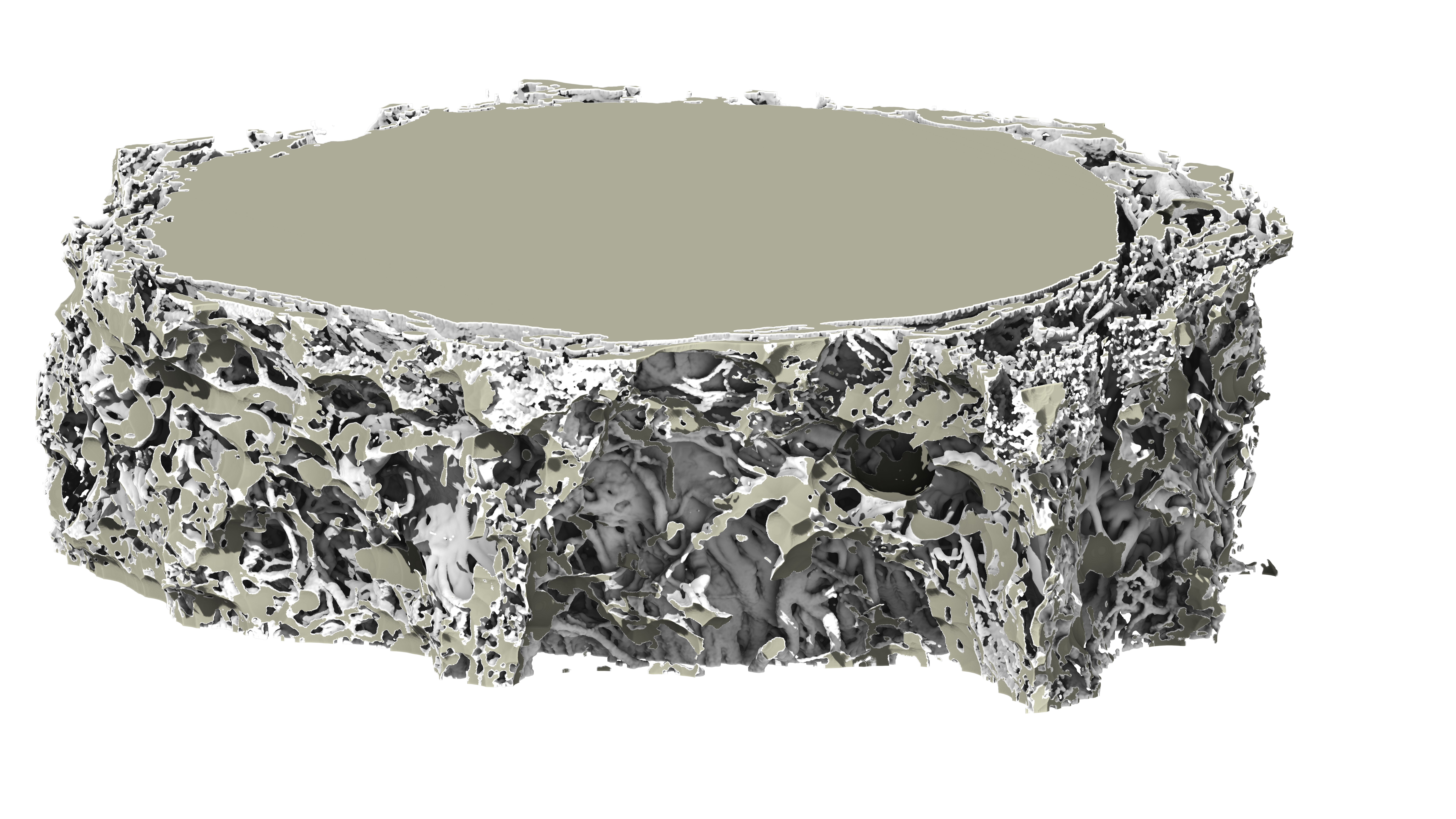

Supplement: Supplementary file 2 — Additional file 2: Overview of the manipulated geometry. [file 12987_2024_518_MOESM2_ESM.zip › S2/4_thicker.png]
