# Supplementary material for: Large-scale in-silico analysis of CSF dynamics within the subarachnoid space of the optic nerve
Source: Fluids Barriers CNS. 2024 Feb 28;21:20. doi: 10.1186/s12987-024-00518-8 (PMC10900650; doi:10.1186/s12987-024-00518-8)
Supplement: Supplementary file 3 — Additional file 3: Validation & Verification of the CFD approach. [file 12987_2024_518_MOESM3_ESM.zip › S3/README.pdf]

CFD : VERIFICATION & VALIDATION

The present document provides details about the proposed CFD approach. It reports on verification and validation studies on a wide range of flow problems, and provides a grid convergence study on the coarsened ONSAS geometry.

ALGORITHMIC & MODELING DETAILS

Reynolds Number

Most flow problems at low Re are expected to reach steady state. Any driving transiency would result in an instantaneous change of the flow field. We expect the flow to remain laminar and fully developed. In the limit case of Re = 0, the convection term disappears --so does the dynamic pressure-- and the Navier-Stokes Equations reduce to a linear system where hydrostatic pressure forces are in balance against viscous forces (Stokes flow). Moreover, we recall that at such low Re the Bernoulli Equation cannot be used to approximate the flow field.

To rule out any doubts about the relevance of the convection term for the specific flow problem at hand, we employ a fully transient CFD solver and carry out the calculations until we reach a steady state. Simulations complete when the temporal difference of the flow fields is below 10^{-6}, in a relative l_∞ sense:

$$\frac{|\mathbf{u}_i^{t+1} - \mathbf{u}_i^{t}|_\infty}{\max(10^{-6}, |\mathbf{u}_i^{t}|_\infty)} < 10^{-6}$$

When comparing the flow on different geometries, we either prescribe a constant hydrostatic pressure gradient, or we enforce a constant volumetric rate. Either way, for all geometrical configurations with the exception of the original one, the Reynolds number will be inevitably altered because the cross-sectional area changes caused by the manipulation.

Discretization

For spatial discretization we rely on high-order finite volume methods on a structured and uniform Cartesian grid. For the temporal discretisation of the Navier-Stokes equation, we rely on the Godunov operator splitting. The boundary conditions at the wall are enforced with an immersed boundary penalty term [Angot 1999]. This model leads to accurate results while providing a simple way of considering complex geometries [Mittal 2005][Rossinelli 2010][Rossinelli 2015]. The model embeds a solid phase (ONSAS structure) within a fluid phase (CSF space) with an implicit description of the geometry based on a signed Euclidean distance transformation. The convection term is discretized by combining a 5th-order weighted essentially non-oscillating scheme (WENO) with Engquist-Osher and Roe numerical fluxes, together with a 3rd-order accurate low-storage Runge-Kutta scheme in time. The diffusion term is solved spectrally (machine precision accuracy) in Fourier space via a Discrete Cosine Transform (DCT) of Type-II/III. The projection scheme is carried out with a 6th-order accurate divergence scheme, a DCT-based spectral Poisson solver, and a 6th-order accurate finite volume gradient. We would like to mention that this discretisation approach is one of the most accurate and efficient that can deal with arbitrarily complex geometries [Rossinelli 2010].

One could argue that high-order DNS is an overly sophisticated approach for flow problems at such low Reynolds number, and a lower order discretization should be used instead. The reasoning behind this argument would be that the numerical dissipation of the associated schemes is tolerable as the flow does not exhibit sharp features. However, the numerical dissipation of low-order schemes is unphysical, as their transfer function is qualitatively very different from the one of a diffusion process. The former notches certain low frequencies and does not attenuate enough some other high frequencies. In turn, a corruption of scales is likely.

Dimensionalization

To minimize numerical issues in DNS, we consider a computational space where finite volume cells feature unit spacing. The dimensionalization is performed by matching the Reynolds number of the DNS with the one of the experimental data (Re_D = 0.1). After quantitative confirmation, the dimensionalization of the velocity is obtained by rescaling velocities (post-processing) consistently with the characteristic velocities of experiment information and simulation. Because of the dominating viscous forces in this flow problem, the nondimensionalization of pressure reads:

$$p' = \frac{pL}{\mu U},$$

where *L* is the characteristic length, *μ* is the dynamic viscosity, and *U* is the characteristic velocity. Accordingly, the relationship between of pressure between simulation and experiments reads:

$$p_{\mathrm{e}} = \frac{U_e}{U_s}\frac{\mu_e}{\mu_s}\frac{1}{h}p_s,$$

where the subscript *e* stands for "experiment" and subscript *s* stands for "simulation", *U* are the characteristic velocities, and *h* is the length covered by a single pixel.

System sizes and computational resources

		System size	CFD working footprint
Dataset1	ROI	3584 x 3968 x 448 cells	-
	Overall	3584 x 3968 x 864 cells	1.5 TB
Dataset2	ROI	3240 x 3240 x 452 cells	-
	Overall	3240 x 3240 x 1280 cells	1.6 TB

Table 1: System size of the high-resolution geometry, and the extended domains.

Table 1 shows the system size and the aggregate RAM footprint of the simulation domains. Each DNS is distributed across the computational nodes of *Helvetios,* without considering the RAM footprint of the external libraries (FFTW-MPI) and runtime (MPI).

Image processing and CFD analysis were carried out on the *Helvetios* supercomputer at École Polytechnique Fédérale de Lausanne (Lausanne, Switzerland). A single DNS run took about 144 hours on 32 nodes for an aggregate nominal peak performance of 35 TFLOP/s in double precision, and a measured aggregate RAM bandwidth of 2.9 TB/s and an all-to-all network bandwidth of 350 GB/s. Overall, more than 10,000 node hours have been spent on the present computational investigation.

COLLAPSE OF A VORTEX RING

Our flow solver is validated against the experimental study by Weigand & Gharib [1] for the collapse of a vortex ring at Re_Gamma = 7500. The present validation also relies on the work of Shariff et al. [2] and the work of Bergdorf et al. [3], for the computational setup as well as for the metrics used in assessing the collected results. Accompanying movies can be found in a subdirectory next to the present document.

[1] Weigand A, Gharib M. On the decay of a turbulent vortex ring. Physics of fluids. 1994 Dec 1;6(12):3806-8.

[2] Shariff K, Verzicco R, Orlandi P. A numerical study of three-dimensional vortex ring instabilities: viscous corrections and early nonlinear stage. Journal of Fluid Mechanics. 1994 Nov;279:351-75.

[3] Bergdorf M, Koumoutsakos P, Leonard A. Direct numerical simulations of vortex rings at ReΓ= 7500. Journal of Fluid Mechanics. 2007 Jun;581:495-505.

Computational setup

On a computational domain uniformly sampled by 1536 x 384 x 384 cells, a vortex ring with outer diameter of 72 cells and inner diameter of 33 cells is placed at ¼ of the domain in the x-direction. The distance between the vortex ring and the lateral periodic boundaries is 155 cells. The CFD was run on 384 cores, in single precision, with periodic boundary conditions. Turbulence decay completed after about 24,100 time steps in about two hours.

The loss of symmetry leading to the vortex ring breakup is caused by imposing 1% random fluctuations in the initial conditions of the flow field, as performed in [2, 3]. To show the robustness of the solver, the present work also includes the DNS of the unperturbed vortex ring.

Qualitative assessment

Throughout the collapse of the perturbed vortex ring we observe the formation of secondary flow structures (hairpin vortices) that induce the ring to a turbulent transition, and a full collapse shortly afterwards. In the unperturbed case, symmetry is maintained almost indefinitely, suggesting remarkable robustness of the employed numerical schemes.

The accompanying movies of the flow field include 8 quantities: the velocity field (u,v,w), the vorticity (omega0, omega1, omega2), and their magnitudes. There are 3 plane-cuts taken in the middle of the domain. The movies include 4 views for line-of-sight orthographic visualizations: 3 from the positive faces of the cartesian domain, and one from the (1,1,1) corner. The movies *vort.mag.corner.vr.mp4*, *vort.1.yside.cut.mp4*, and *vort.mag.zside.vr.mp4* summarize well the evolution of the flow.

Circulation decay

Figure 1 shows the comparison of the experimentally observed circulation decay [1] against the one reported in [3], as well as the decay measured in our CFD results.

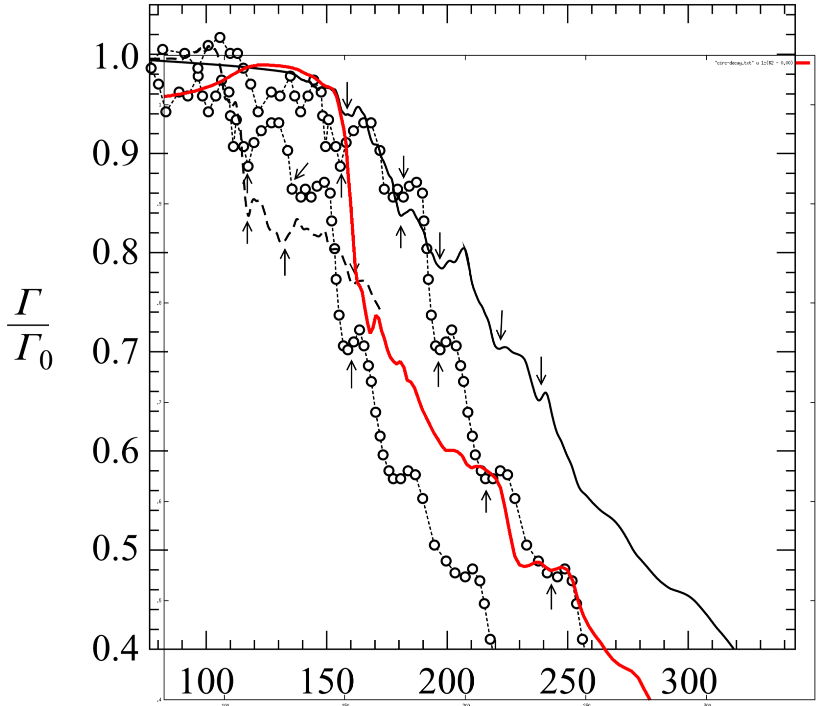

Figure 1: circulation decay of a collapsing vortex ring. Experimental finding [1] (connected circles) are compared against Bergdorf et al. [2] (black curve) and our CFD results (red curve).

Following the same approach of [3], we align our result to the early "circulation drop" of the experimental results [1]. However, we seem to obtain a better matching than [3], both in terms of steepness and steps-like features.

Position Tracking

Figure 2 reports the position of the vortex ring with respect to time, in non-dimensional units, and compares it against the experimental findings. The non-dimensional rescaling of the velocity was performed as in [3].

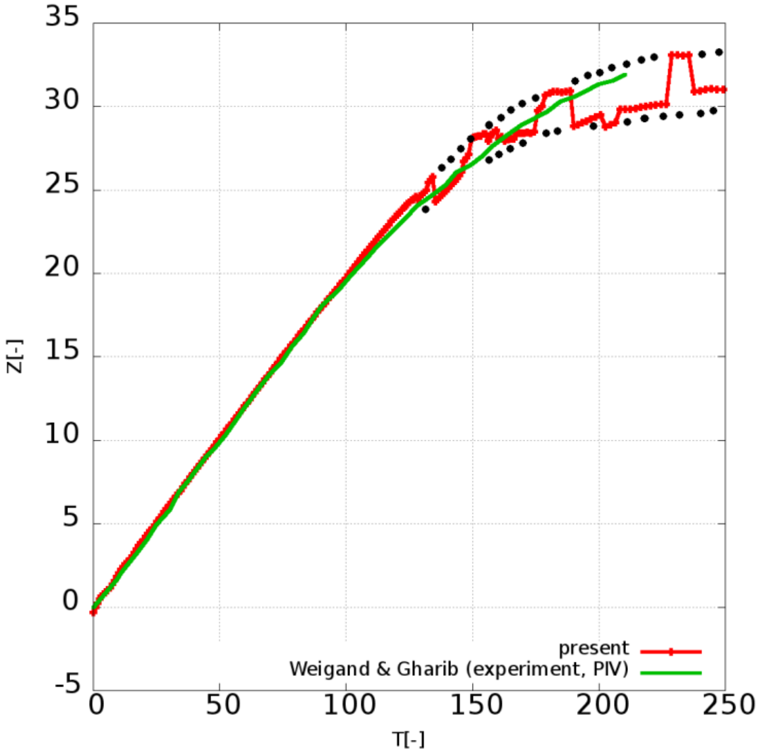

Figure 2: position of the vortex ring over time. Experimental value (green) against our CFD results (red).

Position tracking follows the location of the positive peak vorticity over time. As noticeable in the associated movie, at later stages, transition to turbulence prevents the precise estimation of the position, as sudden peaks are created by secondary vortices. However we can ascertain that the uncertainty interval is centered on the experimental finding, suggesting reasonable agreement also at later stages.

FLOW PAST AN IMPULSIVELY STARTED SPHERE

We report on the accuracy of our approach for the benchmark of the flow past an impulsively started sphere. The assessment is carried out qualitatively against the experimental work of Taneda [1], Sakamoto & Haniu [3], and quantitatively against the work on Roos [2], Sakamoto & Haniu [3]. This flow problem is very challenging, and a complete investigation would deserve a dedicated paper. The goal of the present validation is thus limited to the goals summarized by Figure 3: we show that our CFD results are sound, and show that the obtained flow fields are reasonably well aligned with the experimental results, both qualitatively and quantitatively.

[1] Taneda S. Experimental investigation of the wake behind a sphere at low Reynolds numbers. Journal of the physical society of Japan. 1956 Oct;11(10):1104-8.

[2] Roos FW, Willmarth WW. Some experimental results on sphere and disk drag. AIAA journal. 1971 Feb;9(2):285-91.

[3] Sakamoto, Haniu, and H. Haniu. "A study on vortex shedding from spheres in a uniform flow." (1990): 386-392.

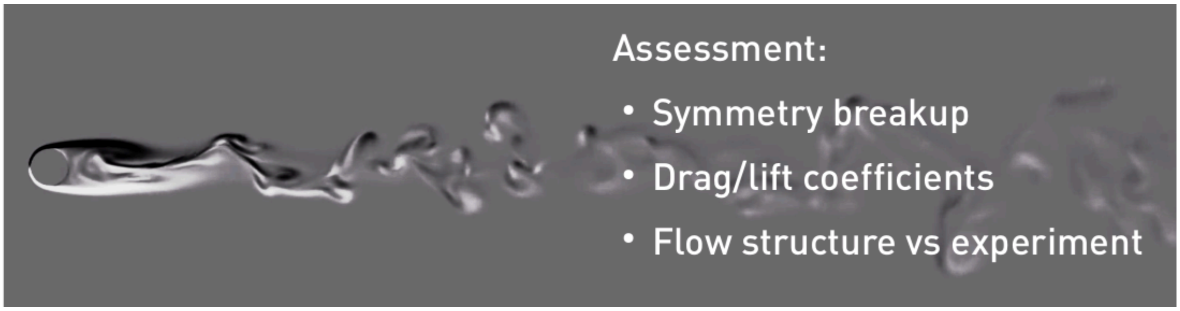

Figure 3: Vorticity field (component normal to the visualized plane) for Re = 1000. The present flow benchmark demonstrates the numerical robustness of our schemes, in terms of late breakup of symmetrical flow structures, a qualitative comparison of the vortical structure, and a quantitative assessment of the drag coefficient.

Computational Setup

A flow past an impulsively started sphere is considered at Re_D = 300, 500, and 1000, with CFD simulations in double precision consisting of 2560 x 512 x 512 cells, and approximately 50,000 timesteps, with a CFL of 0.3. We assume zero-Neumann conditions for the velocity field at the domain boundaries, and no-slip no-through conditions at the wall boundaries. The geometry is sampled with about 60 cells over the sphere diameter, and for the immersed boundary term we use a mollification length of 3 cells. The sphere is placed downstream at ⅕ of the computational domain from the inlet, and the distance between the sphere and lateral boundaries is 4 diameters. No perturbation was injected in the flow field.

Technical issues arise from the fact that the sphere must be resolved well enough to capture the detailed vorticity that accounts for skin friction, while ensuring that the computational boundaries are distant from the sphere in order for the latter to not feel the effect of the artificial boundary conditions, both upstream and laterally. Moreover, the domain should be long enough downstream to cover the strongest part of the trailing wake, as the latter is the main contributor to the drag coefficient. Such requirements, alongside with the timespan required to reliably sample vortex shedding, make this benchmark very computationally demanding and still represent a grand challenge in CFD to date.

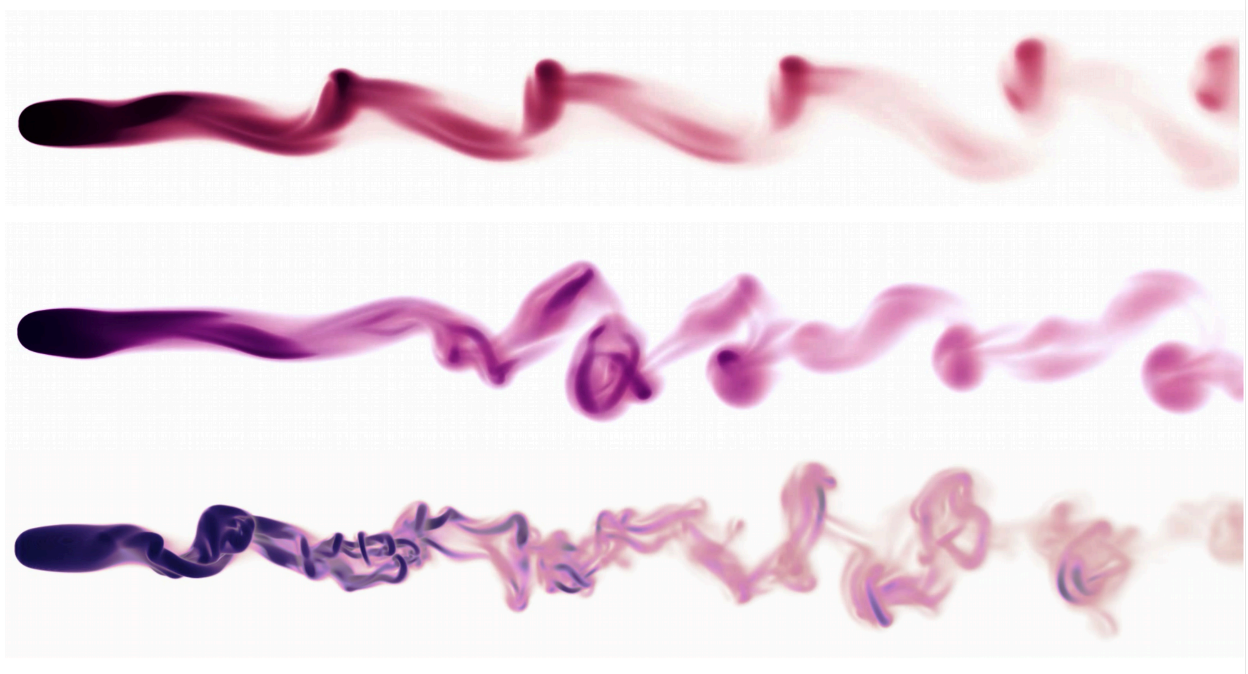

Figure 4: Line-of-sight visualization of vorticity magnitude for Re = 300 (top), 500 (middle), and 1000 (bottom), respectively.

Qualitative Assessment

Figure 4 illustrates the shape of the trailing wake with respect to the Reynolds number, after vortex shedding has established. Increasing the Reynolds number leads to progressively sharper flow structures and a systematic emergence of small-scale details.

The accompanying movies are informative for different reasons. Firstly they convey how enduring the flow structure symmetries are, before vortices are shed from the body. Secondly, movies show that after a short time interval, vortex shedding consistently exhibits specific frequencies as experimentally

observed (related to the Strouhal number). Thirdly, as summarized by Figure 5, the numerical flow field is in good qualitative agreement with the experimental study of Sakamoto and Haniu [3]. Fourthly, the conceptual flow structure depicted in Figure 5 (left) are well matched by the structures visualized in our snapshot movies.

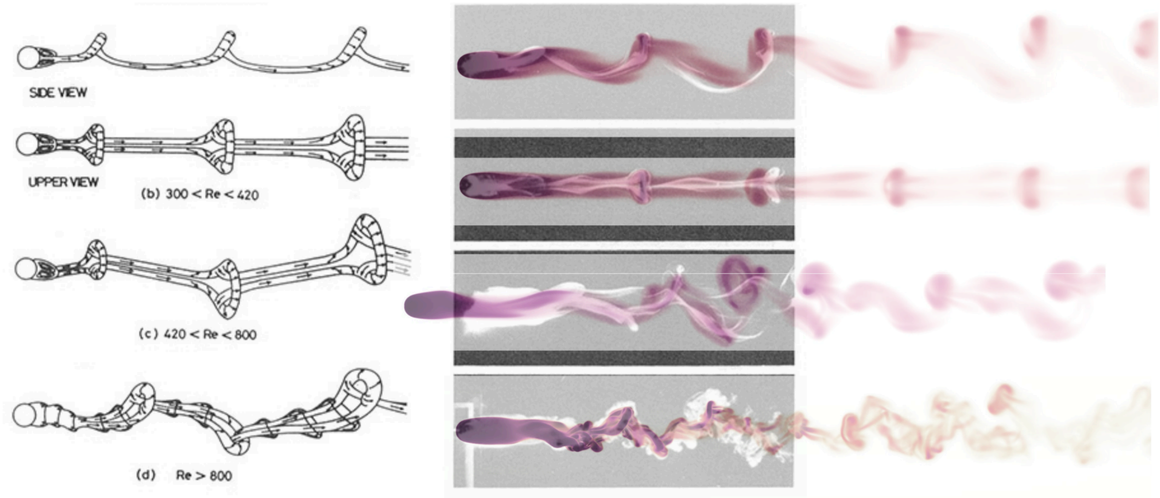

Figure 5: Conceptual sketch of the vortical structures in the trailing wake (left) taken from [3], and qualitative comparison of the trailing wake between experiments [3] (grayscale picture, right) and our CFD results (violet).

Quantitative Assessment

The drag coefficient from our CFD results is compared against the experimental quantities reported by Roos [1] and Sakamoto & Haniu [3]. Figure 6 depicts the drag coefficient with respect to time, for Re = 300, and 500, respectively. The impulsive nature of the sphere start is clearly noticeable at early stages and covers a substantial fraction of the physical time. For Re = 300, we observe a discrepancy in the drag coefficient of about 5% (experimentally 0.68, numerically 0.72, respectively), when considering the average value in [3] and compare it against the shedding configuration. At Re = 550, we observe a larger discrepancy of about 13% (experimentally 0.55, numerically 0.63, respectively).

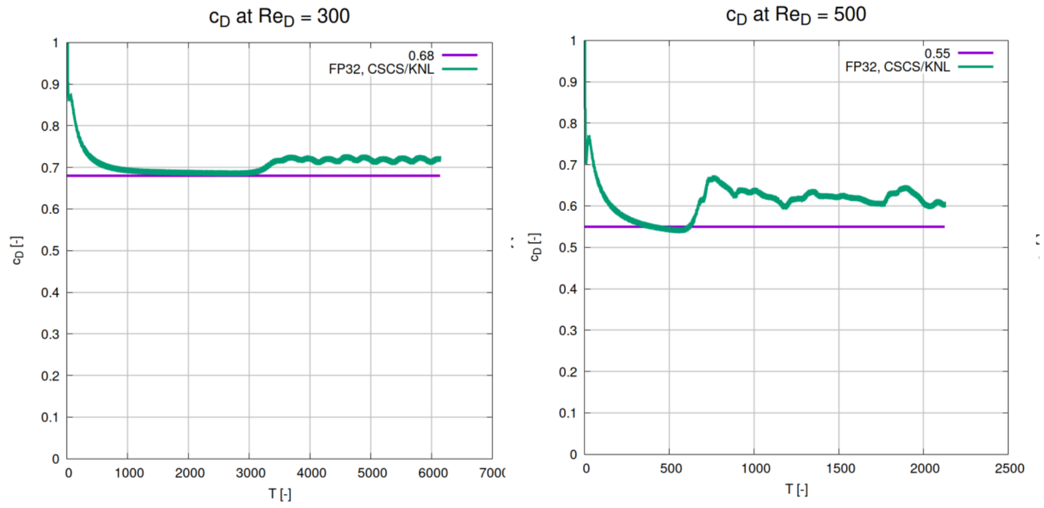

Figure 6: Drag coefficients for Re = 300 (left) and Re = 500 (right), from the experimental investigations in [2] and [3] (violet) averaged over time, and from our CFD results (green).

Figure 7 reports the evolution of the drag coefficient for Re = 1000. The overprediction of the drag coefficient of our DNS seems to worsen by increasing the Reynolds number, as we observe a relative discrepancy of 16% between experimental and numerical findings. The worsening in accuracy is to some extent expected as we keep the same spatial resolution while we increase the Reynolds number, and therefore we fail to accurately capture the emergence of smaller scales at the wall. Moreover, the inertial forces at this Reynolds number seem strong enough to inject chaotic traits in the flow. This is demonstrated by deviations in the drag coefficient by carrying out CFD with the bitwise identical initial conditions, same computational schemes (identical source code), and instruction streams targeting different microarchitectures (AVX, AVX2, and AVX-512).

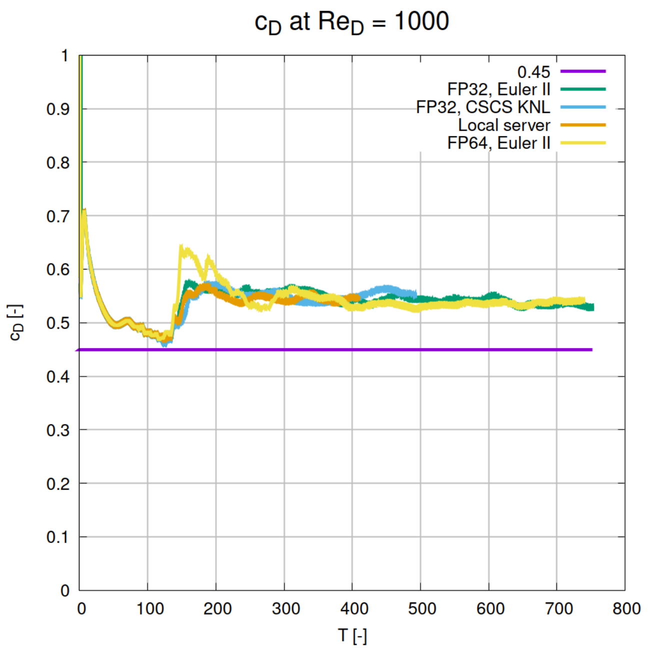

Figure 7: Drag coefficient over time for Re = 1000. The same simulation leads to slight differences depending on the computing platforms where the CFD is carried out.

FLOW IN A CURVED PIPE

As a benchmark, we reproduce the velocity profiles of curved pipe flows reported by Verkaik et. al [1]. We do this by using the same binary executables used for the ONSAS CFD. Accompanying movies are included in the same directory of the present document.

[1] Verkaik AC, Beulen BW, Bogaerds AC, Rutten MC, van de Vosse FN. Estimation of volume flow in curved tubes based on analytical and computational analysis of axial velocity profiles. Physics of fluids. 2009 Feb 1;21(2).

Computational Setup

We consider a system size of 360 x 360 x 360 cells in a cubic computational domain, and capture the geometry of the coiled pipe shown in Figure 8. In their work, Verkaik et al. considered an idealized curved pipe which would unphysically intersect itself. For our CFD we consider a realistic geometry which is also exposed to discretization errors, as visible in the figure.

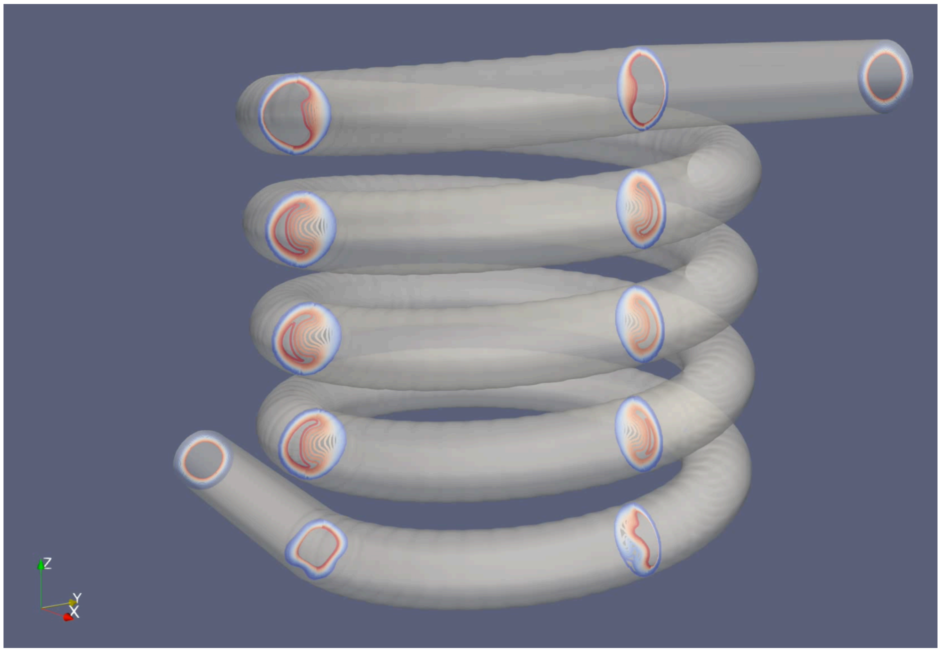

Figure 8: Geometry of the coiled pipe, visualized with flow speed isocontours from our CFD results.

CFD was carried out in double precision on a single compute node, with a CFL of 0.05 and viscosities to match Dn=680 and Dn=10, respectively. The Brinkman penalization parameter was set to 5E-5, and the mollification length to capture the flow-structure interface covered 3 grid points.

Quantitative Assessment

We compare the velocity profile in the same fashion as proposed in Figure 7 of [1].

Dn = 680

As shown in Figure 9, we obtain very good agreement to the profiles reported by Verkaik et al., both along the axis of symmetry of the cross-sectional plane, as well in the orthogonal direction.

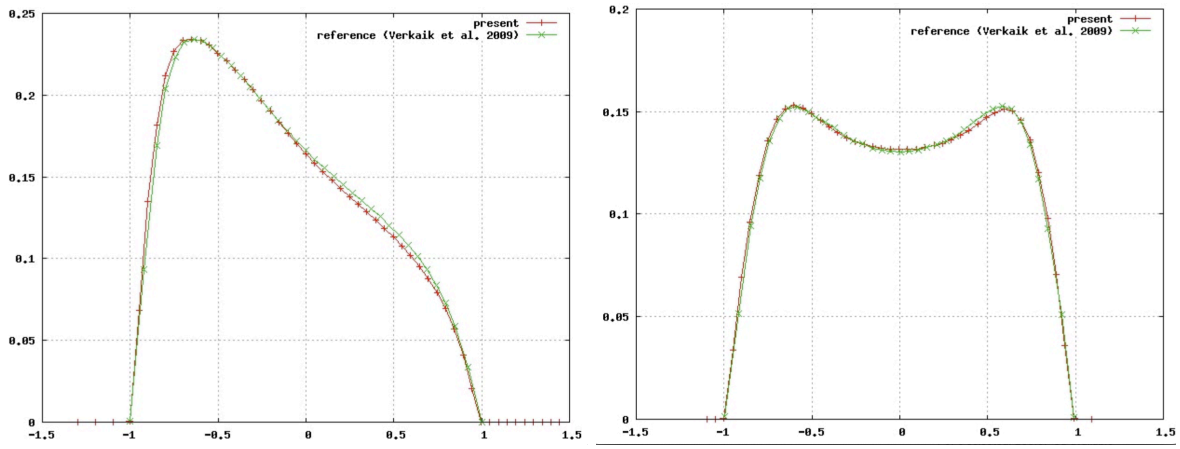

Figure 9: velocity profiles for the Dn=680 benchmark by Figure 7 of [1] (green) and our CFD results (red), along the axis of symmetry (left) as well as in the orthogonal direction (right).

Dn = 10

We experienced some difficulties in digitizing the reference curve for Dn=10: In Figure 7 of [1] the associated signal has a very low peak. Nonetheless, our results and the reference profile remain in strong agreement, as shown in Figure 10. At this flow regime we observe an axisymmetric velocity. Accordingly, we provide a single velocity profile.

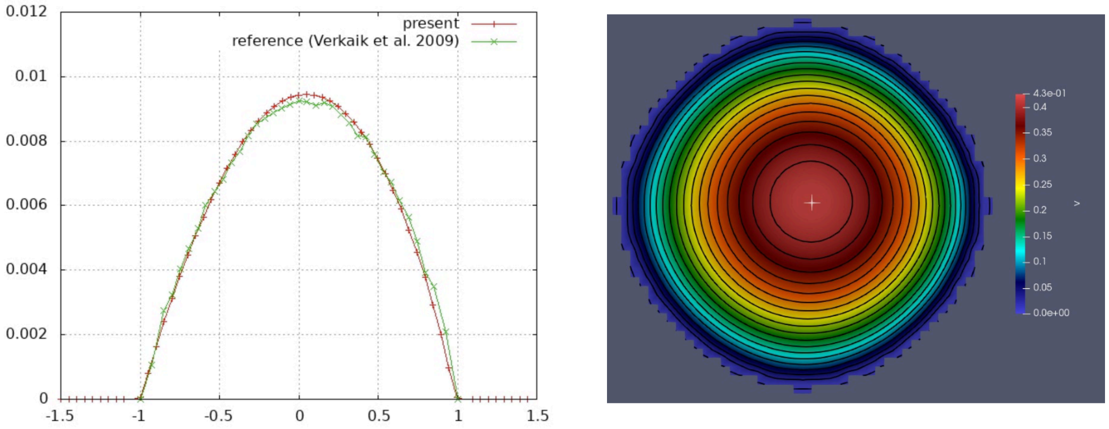

Figure 10: Velocity profile for the benchmark flow problem at Dn=10 (left) of [1] (green) and our CFD results (red), and flow speed in the cross-sectional plane (right).

Pressure field

Since [1] does not provide quantitative data on pressure, we rely on a qualitative comparison. Whenever we want to compute the total pressure, we solve an additional Poisson equation:

$$\Delta p = \nabla \cdot [\nabla \cdot (\mathbf{u}\mathbf{u}^\top)]$$

We rely on the same computational schemes used to discretize convection and projection terms.

Figure 11 shows a one-to-one qualitative comparison of the pressure reported in [1] against our pressure. The slight tilting of our result is due to the realistic pipe we are considering, as opposed to idealized, and is proportional to the coiling slope of the geometry.

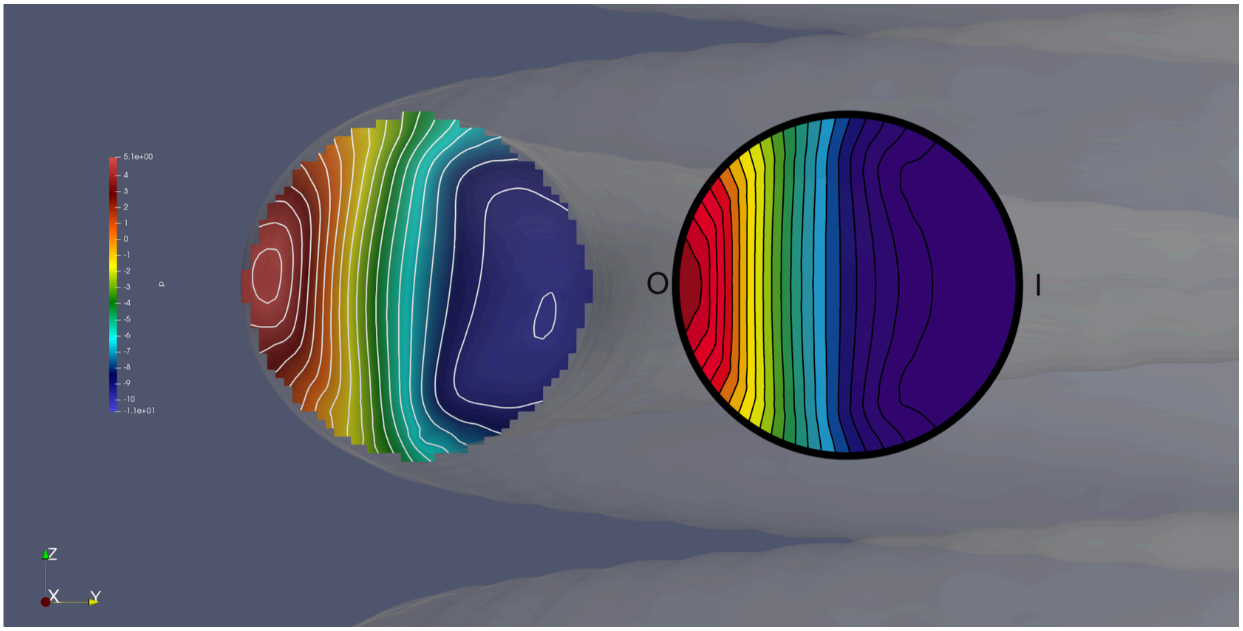

Figure 11: visualization of the pressure profile of our CFD results obtained on realistic geometry (left) and the one reported in [1] based on an idealized geometry (right).

CFD ON COARSE ONSAS GEOMETRY

We report on the spatial convergence for a Poiseuille flow at Re = 0.1, starting from fluid at rest. Convergence is examined both for steady-state flow and while flow transitioning to steady state. Moreover, spatial convergence is examined for a flow within the ONSAS geometry.

Poiseuille flow

Starting with the fluid at rest, we impose a hydrostatic pressure gradient across a circular cylinder. The pipe is modeled with no-through and no-slip boundary conditions. By matching the Reynolds number = 0.1, we consider the same geometry at different resolution, with the following system sizes:

- 80 x 80 x 160 cells,
- 120 x 120 x 240 cells,
- 180 x 180 x 360 cells,
- 270 x 270 x 540 cells, and
- 405 x 405 x 810 cells.

Throughout time, the flow fully develops, leading to a smooth and stationary velocity profile. This is exemplified in the movie *typical-profile.mp4,* reported in simulation (i.e. dimensional) units.

Steady-state Assessment

When the simulation achieves steady state, numerically defined as mentioned above, we measure the flow speed at each point of the cross-section in the middle of the pipe (including the solid phase), and compare it against the analytical solution. Figure 12 shows *relative* l1 error norms for the considered grid spacing. The resulting order of convergence is approximately quadratic.

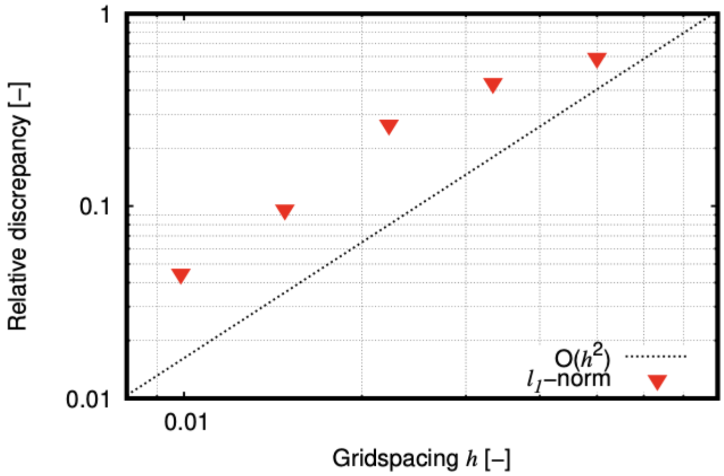

Figure 12: Relative error between our steady-state CFD and the analytical solution of the Poiseuille flow.

Transiency Assessment

We investigate spatial convergence while the flow has not yet reached steady state. The assessment is carried out when the peak flow speed is approximately 1⁄5 of the steady-state one. Since we are unaware of analytic solutions for the flow field at this instant, the verification is performed by comparing the coarser results against the results of the finest grid. Accordingly, we consider additional system sizes:

- 607 x 607 x 1214 cells, and
- 911 x 911 x 1822 cells.

The CFD simulation with 911 x 911 x 1822 cells is considered as a reference solution to quantify distortion. The latter is reported in Figure 13, by spectrally resampling the coarser velocity field to the finest resolution. We observe that in this regime convergence is first order. The order of convergence is lower than for steady-state (Figure 12), possibly because the contribution of the immersed boundary term is larger during transiency, and it is known to be first order accurate.

The particular ranking of the norms in Figure 13 suggests that errors accumulate at specific locations of the domain, rather than uniformly everywhere.

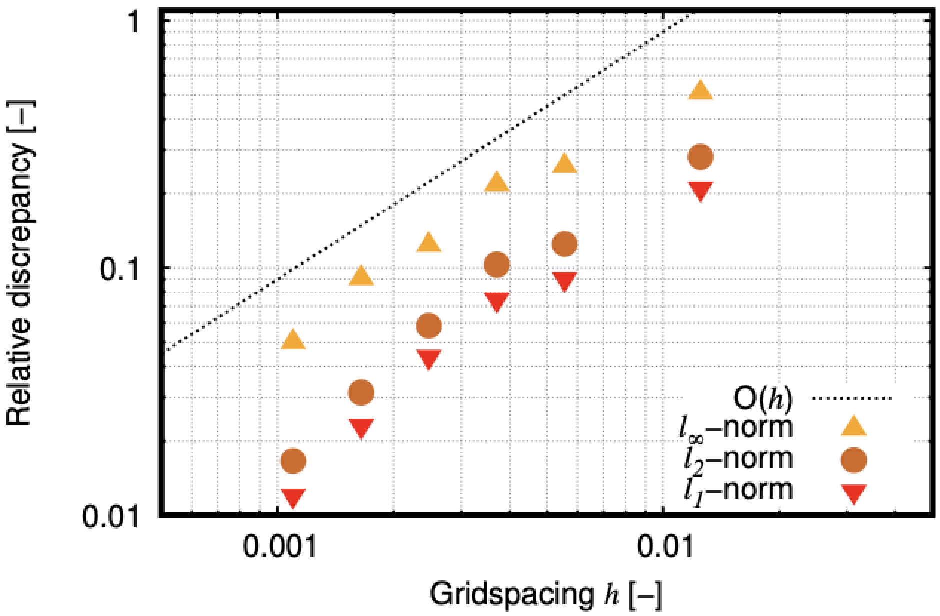

Figure 13: CFD at different grid spacing are compared against the simulation at the finest resolution.

Coarse ONSAS Geometry

We coarsen the geometry considered in the manuscript from 3584 x 3968 x 864 samples down to 224 x 248 x 54 samples. The downsampling is carried out spectrally with DCT-II/DCT-III, and is preceded by a Lanczos kernel-based low pass filter, to eliminate all geometrical details that the CFD system size cannot correctly resolve. The geometry is then upsampled for CFD purposes, with the system sizes reported in Figure 14.

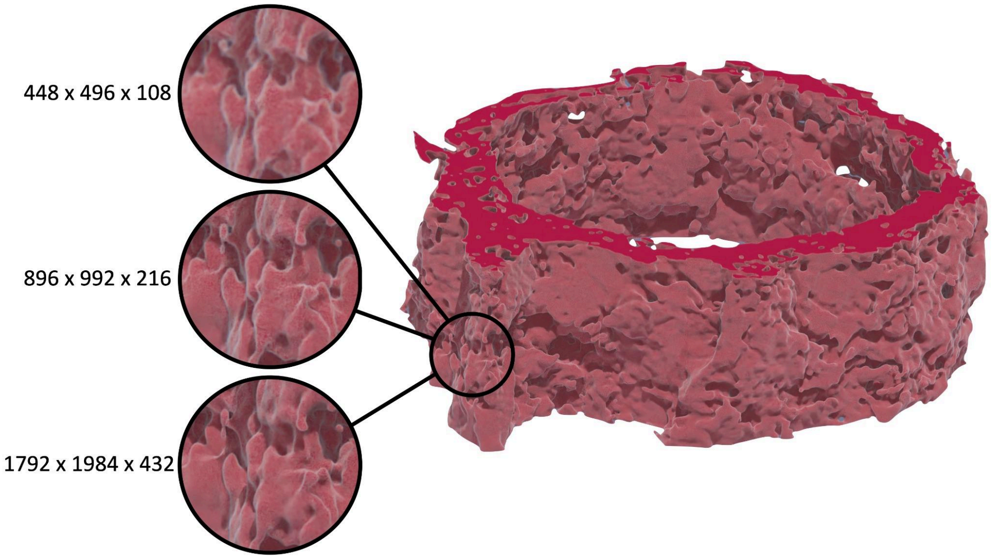

Figure 14: Upsampling of the geometry for CFD spatial convergence purposes, from a signal sampled with 224 x 248 x 54 grid points.

The absence of analytical solutions for this geometry forces us to define a distortion in terms of a highest-resolution CFD consisting of 3584 x 3968 x 864 cells. To compute distortion, coarser simulations are resampled spectrally to the finest resolution. Figure 15 shows superlinear convergence.

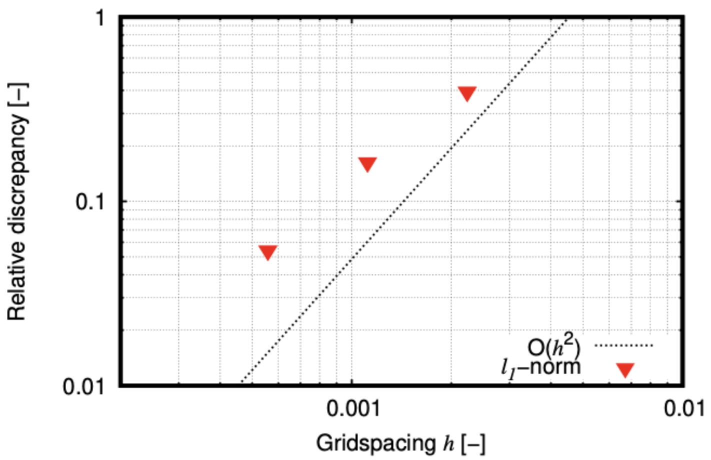

Figure 15: Distortion of CFD results at different grid spacing, against the finest CFD resolution.
